# Uncertainty Propagation and Input Sensitivity in Life Cycle Assessment: An Application to Phase Change Materials

**DOI:** 10.1021/acssusresmgt.5c00298

**Published:** 2025-08-18

**Authors:** Humberto Santos, Silvia Guillén-Lambea

**Affiliations:** Aragón Institute for Engineering Research (I3A), Thermal Engineering and Energy Systems Group, 16765 University of Zaragoza, Agustín de Betancourt Building, C/María de Luna 3, Zaragoza 50018, Spain

**Keywords:** life cycle assessment, Monte Carlo sampling and uncertainty propagation, sensitivity analysis, SALib, midpoint indicators, end point indicators

## Abstract

Global and local sensitivity analyses are essential for identifying key parameters in life cycle assessment models. However, due to limited information on parameter uncertainty, they are often overlooked. This paper’s objective is to address this gap by proposing a methodological framework for defining input sensitivity, for midpoint and end point indicators, and a quantitative approach for determining input uncertainties. Applied to a case study on xylitol production as a phase change material, the methodology uses Monte Carlo for uncertainty propagation and Python’s SALib to calculate Sobol indices. Results show a 2% relative error in midpoint indicators, aligning with pedigree matrix methods. While accuracy depends on choosing the appropriate distribution function, both global and local sensitivity analyses showed consistent outcomes. This structured, user-friendly approach offers decision-makers a simplified yet effective way to prioritize inputs, either by verifying multiple indicators individually or focusing on damage-oriented indicators. Future studies could refine database coefficients and explore their influence on overall uncertainty, as well as the nonlinearity of the model if the parameters are correlated, offering opportunities to enhance accuracy.

## Graphical abstract

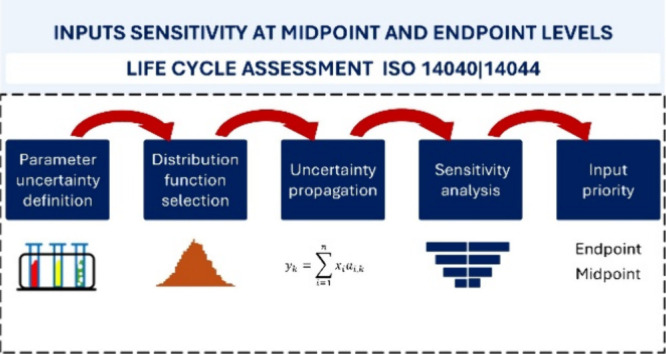

## 1 Introduction

Life cycle assessment (LCA) is crucial for evaluating the environmental impacts of products and services. In energy storage, it has assessed solar-based energy storage systems,^1^ and phase change material (PCM) from cradle to grave.^2^ However, LCA is inherently uncertain^3^ owing to data variability, procedural assumptions, and data errors.^4^ In response to this difficulty, various studies propose methodologies to treat data quality, parametric uncertainty, uncertainty propagation, and sensitivity analysis. Nonetheless, a search in the Scopus database of studies focusing on LCA of PCMs published between 2020 and 2024 revealed that almost 80% of 44 studies did not include uncertainty or sensitivity analysis. This reflects the difficulties faced in integrating uncertainty and sensitivity analysis in LCA. Therefore, addressing data uncertainty and its propagation from inputs to outputs is essential to improve LCA robustness.

Incorporating data quality in terms of parameter uncertainty is a common challenge when carrying out LCA across various fields. Studies show that few LCA investigations report data uncertainty.^5^ Addressing uncertainties within LCA is difficult due to the complexity of many interconnected variables^6^ and of inability to verify, validate, or confirm results.^7^ Environmental assessment results also vary when converting inventory flows into indicators, with LCIA methods contributing to uncertainties due to emission values, substance coverage, and characterization factors.^8^ ISO 14040/14044 guidelines recommend considering uncertainty in terms of time, geography, technology, and uncertainty of the data, to name a few.^9,10^ To meet these requirements, the pedigree matrix, originally developed for “all sorts of uncertainty”,^11^ was implemented in the studies of environmental assessment,^12^ and remains the most widely used method for defining qualitative data uncertainties in LCA research.

Several scientific publications address uncertainty and sensitivity analysis, proposing methodologies to undertake this task in LCA. A detailed review of the most notorious works is provided in the Supplementary Material. Geisler et al. developed a methodological approach for uncertainty analysis in LCA using equations to estimate best – and worst-case flow scenarios.^13–15^ Wei et al. (2015) applied matrix-based LCA theory for uncertainty propagation and sensitivity analysis in the inventory phase.^16^ The literature accounts for multiple methodologies for treating data uncertainty in LCA (Cucurachi et al., 2016; Di Lullo et al., 2020; Qiao et al., 2025; Shi and Guest, 2020). This study introduces a five-step framework to aid LCA practitioners in identifying parameter uncertainty, selecting distribution functions, and propagating uncertainty to outputs via GSA and LSA. It focuses on the LCIA phase, benefiting users of multiple software-integrated databases. It extends input sensitivity analysis beyond midpoint indicators to end point levels, providing deeper insights into input significance in LCA models from a damage-oriented perspective. Table 1 compares the characteristics of previous studies with the present work. A valuable tool for implementing GSA is the sensitivity analysis library written in the Python programming language, known as SALib, which offers various sampling and sensitivity analysis methods.^21,22^ However, the use of this tool has been limited to a small number of studies. On polygeneration systems, SALib was used to investigate the impact of scale-up and uncertainty in economic parameters, such as fuel cost as a function of engine type, on the products in the system.^23^ Most recently, Stajić et al. (2024) used this library to investigate the effect of parameters on natural gas prices in a global international market. They calculated Sobol indices to quantify the importance of each parameter’s uncertainty in the overall uncertainty of the outputs. This approach facilitated the comparison of influencing factors and demonstrated that global natural gas prices are most significantly affected by crude oil prices.^24^ In building energy performance optimization, for instance, distinct parameters were investigated to understand their impacts on the heating and cooling of buildings in Morocco.^25^

**Table 1:** Comparison between the Previously Published Papers and the Present Work Methodologies^a^

Application	Uncertainty type	Probability distribution	Sensitivity type	LCIA method	Ref
Plant-protectionproducts	Quantitatively derived by comparing LCIs	Lognormal for most parameters, it only yields positive values	Not reported	CML-baselineSelected Midpoint indicators	^14^
Glass wool mat	Uncertainty data from Ecoinvent.	PDF from Ecoinvent. Lognormal PDF is used	Dependent and independent GSA are done in R Software	IMPACT2002+ Midpoint and end point indicators	^16^
Noise on human health	Uncertainty is informed as SD accompanied by the probability distribution function of each parameter	The PDF is selected based on expert knowledge or published data. PDF is identified, and a sample is generated for inputs. The uncertainty is then propagated to the outputs.	GSA is applied to this study using the R Software	Specific equations are used to calculate the defined noise model impact categories indicators	^17^
Conventional Crude Oils and Oil Sands	The uncertainty of parameters is provided in the form of maximum and minimum values	Lognormal, normal, PERT, triangular, and uniform can be used	GSA and regression using Regression, Uncertainty, and Sensitivity Tool (RUST)	Calculation using a single-issue method. GHG emissions have been described	^18^
Ethanol Production from Sugarcane	Uncertainty of all inputs and outputs is empirically based on uncertainty factors using the Pedigree Matrix	The majority of exchange flows have a log-normal probability distribution predefined.	GSA is used to investigate how sensitive the inputs are to the outputs	TRACI v2.1 has been used to obtain midpoint indicators	^20^
Hot and cold recycled asphalt pavement	Uncertainty values are derived from the Pedigree Matrix as data quality scores	Beta distribution functions	LSA is used to identify influential inputs as the first step	CMLMidpoint Indicators	^19^
Bio-based phase change material	Quantitative uncertainty is derived from the production process and then compared to the most common uncertainty scores used currently	This work uses a function check step to select the best PDF for the input parameter. The check to validate the PDF is done in the input uncertainty propagation step. Only if the function is suitable, the uncertainty is propagated to the outputs (LCA indicators)	LSA and GSA using SALIB written in Python. This is done at the LCIA phase. Apart from discussing the sensitivity of midpoint indicators, this work also gives insights into how sensitive inputs may impact end point indicators, with both LSA and GSA.	ReCiPe 2016 Midpoint (H) and End point indicators	This work

a Note: PDF – probability distribution function; SD – standard deviation.

Given the exposed scenario, this work’s objectives are twofold. First, it proposes a five-step methodological procedure for propagating uncertainty and determining input sensitivity at problem-oriented and damage-oriented levels, and compatible with any LCIA method. Second, it proposes a quantitative approach for defining the parameter uncertainty of LCA model inputs for a specific case study, compared with two conventional approaches. A review of the published works is conducted, especially for the case of previously developed frameworks for sensitivity and uncertainty treatment in LCA (Supplementary Material). Monte Carlo sampling and local and global sensitivity analyses are used, the latter through SALib. A case study of a bio-based PCM consisting of the production of xylitol is addressed due to its potential application in thermal energy storage. This work contribution relies on a procedure for propagating uncertainty in LCA studies, extending the discussion of uncertainty and sensitivity analyses to end point indicators, such as human health, ecosystems, and resources, unlike most studies that focus on midpoint indicators. It offers practical implications for propagating uncertainty in bio-based PCMs and LCA studies, particularly in the thermal energy storage sector, however, any LCA study can benefit from the procedure presented since it is not limited to this field of study. This methodology aids decision-making on the environmental impacts of new PCMs in ongoing research.

## 2 Materials and Methods

### 2.1 Case Study

This study follows ISO 14040 and 14044 LCA guidelines.^9,10^ An LCA case study consisting of the production of xylitol is considered, having 1 kg at the factory’s gate as the functional unit (FU). Xylitol was chosen for this study due to its potential as a bio-based PCM. Derived from lignocellulosic biomass, more specifically from the hemicellulose,^26^ xylitol’s nature itself presents an advantage over fossil-fuel-based PCMs. However, LCA input uncertainty remains a key theme, due to limited information regarding the variation of the parameters involved in the production of this PCM. Xylitol is a bio-based substance manufactured from distinct biomasses. Lignocellulosic biomass contains mainly three components: cellulose, hemicellulose, and lignin, from which hemicellulose is the source of xylose, subsequently turned into xylitol.^27^ The yield of xylitol is contingent upon the hemicellulose content of the feedstock, which is determined through prior laboratory characterization of its properties. This hemicellulose content can vary even within the same type of lignocellulosic material. In sugarcane, hemicellulose content (*m*_*hemi*_) varies in range 25-35%. For the production inputs, the amount of sulfuric acid used in the biomass digestion is defined by the relation *m*_*H*2*SO*4_ = 0.45*m*_*hemi*_,^28^ which will return distinct H_2_SO_4_ values depending on the hemicellulose content previously verified for the feedstock. Consequently, the quantity of other inputs changes accordingly, such as water for acid dilution and lime. Numerous studies have been carried out regarding the use of xylitol as a phase change material.^29–31^ Xylitol has a latent enthalpy of 240 kJ.kg^–1^ and a melting temperature of 93°C.^32^ For instance, the energy content of xylitol is almost two times higher than magnesium nitrate hexahydrate, Mg­(NO_3_)_2_.6H_2_O, used as PCM for nearly the same range of temperature application.^33^ Moreover, sugar alcohols contain more than twice the energy density of other PCMs, such as paraffin wax.^30^ Additional explanation regarding the LCA phases and the inventory can be found in Section S1 and Table S1, respectively, of the Supplementary Material. The boundary system for the inputs considered in the manufacturing stage is depicted in Figure S1 of the Supplementary Material. While this methodology focuses directly on the thermal energy storage sector, it can also be extended to other studies that include life cycle analysis with more or fewer modeling parameters, thus allowing the propagation of uncertainties and the sensitivity analysis of its parameters with a focus on decision-making.

### 2.2 Uncertainty and Sensitivity Methodological Structure

Additional details on the methodological steps adopted in this study are presented and illustrated in Figure 1. From the left, it shows the LCA phases that are carried out simultaneously with the proposed approaches. On the right side, the methodology is organized into five main steps: parameter uncertainty definition; distribution function selection; uncertainty propagation; sensitivity analysis, and input sensitivity. Usually, parameter uncertainty is not known for LCA studies and must be considered to understand how the inputs and outputs behave within a specified range. In this work, uncertainty is included on a quantitative basis for the PCM being under study and compared to other approaches commonly used in the literature. A detailed review of the methodologies developed by other authors is presented in the Supplementary Material. Knowing the range or standard deviation (SD) of a parameter is not enough, and sampling with stochastic methods is necessary. This comprises step 2 of the methodology. Then, a mathematical expression is established to propagate the uncertainty to the indicators, defined in step 3. LSA and GSA are used to investigate the behavior of input variation and statistical influence on the outputs, presented and discussed in steps 4 and 5, respectively. Apart from these, all LCA phases are coordinated in parallel and interrelated.

**Figure 1:**
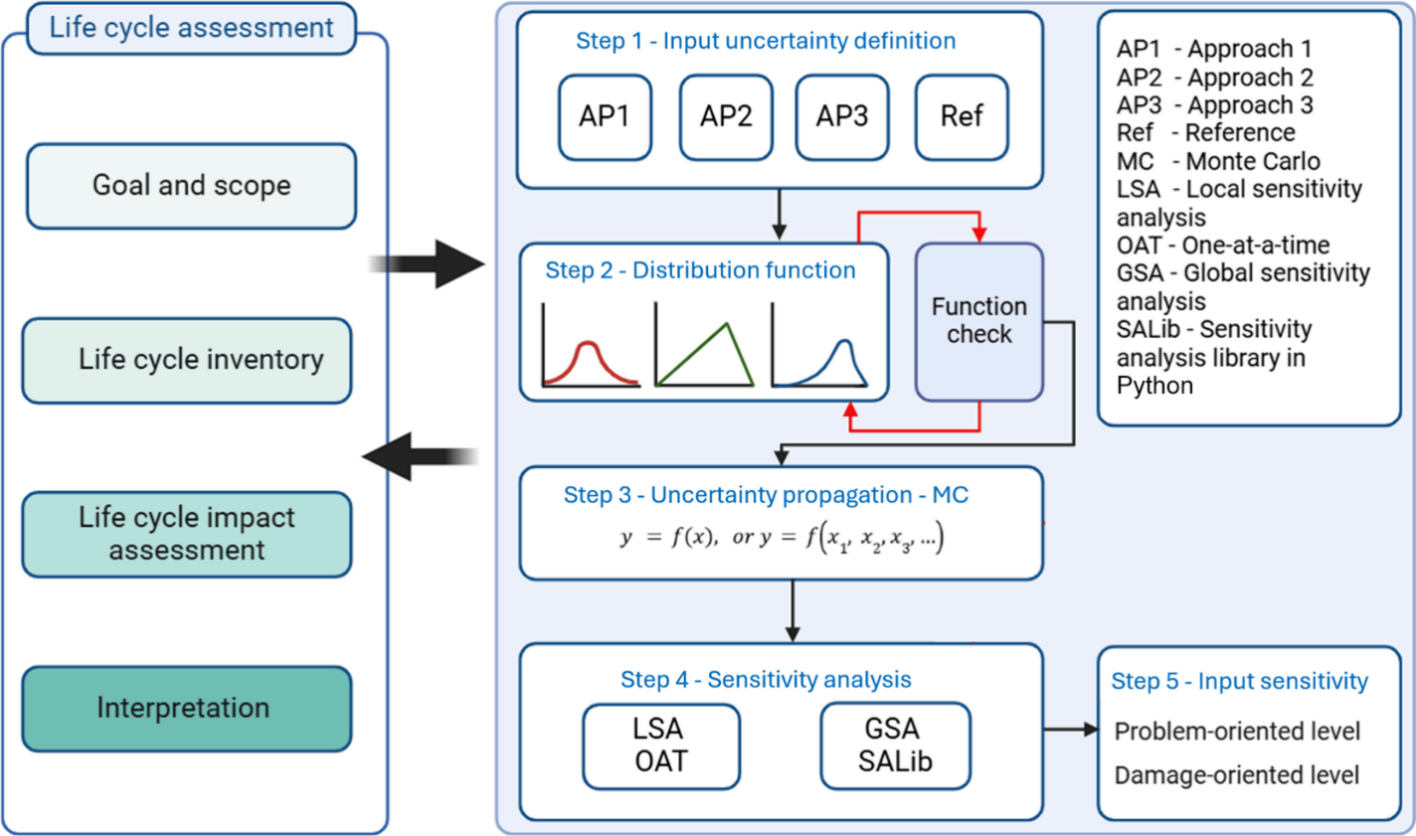
Five-step methodology structure for propagating uncertainty and determining input sensitivity at mid and end point levels.

In step 1, the parameter uncertainties of the inputs are derived. Three approaches are considered in this investigation such as approach 1 (AP1), approach 2 (AP2), and approach 3 (AP3). AP1 is the quantitative approach proposed in this study, which is then compared to AP2 and AP3. The parameter uncertainty in this work regards numerical information related to the statistical variability of parameters, in this case, either the upper or lower bounds or the standard deviation of an input. Apart from the three approaches, the results are constantly contrasted to the reference case (Ref), which is the condition of unknown parameter uncertainty obtained directly from SimaPro using Ecoinvent.

AP1 - This approach proposes to derive the uncertainty ranges (upper and lower bounds of the inputs) based on the PCM production process, which thus has a quantitative nature. To clarify this approach, a more comprehensive understanding of the production process of this PCM is required (Section 3.1). This approach is primarily proposed because the process design for xylitol production and the associated material requirements should ideally be based on the precursor content, specifically the xylose. The description of each input’s variation is given in the Supplementary Material (Table S2). They were obtained with the explanation presented in Section 3.1 regarding the xylitol production process. The range of variability for this approach is shown in Table 2. Besides xylitol, many other bio-based PCMs obtained from lignocellulosic biomass can have their uncertainty derived in the same manner, such as erythritol and bio-adipic acid,^34^ or plant oil-based PCMs like jojoba oil and coconut.^35^

**Table 2:** Parameter Uncertainty According to the Methodology

			AP1		AP2		AP3
Input	Variable	Ref	Min	Max	Min	Max	SD
Sugarcane bagasse	x_1_	10.469	8.106	11.348	6.281	14.657	1.218
Water	x_2_	45.868	35.560	51.680	29.814	61.922	1.219
Sulfuric acid	x_3_	1.347	1.100	1.600	0.875	1.818	1.219
Lime	x_4_	1.055	0.860	1.250	0.686	1.424	1.219
Hydrogen	x_5_	0.026	0.020	0.028	0.017	0.035	1.219
Raney-nickel	x_6_	0.038	0.029	0.041	0.025	0.051	1.219
Electricity	x_7_	1.282	0.993	1.390	0.833	1.731	1.219
Steam	x_8_	16.744	12.965	18.150	10.884	22.604	1.219

AP2 - Parameter uncertainty was derived from the pedigree matrix, currently the most used source of uncertainty indicators for LCA data.^12^ It consisted of a data quality assessment (DQA) from which data quality indicators (DQI) were obtained for each input, with a subsequent aggregation of these data quality indicators (ADQI). Then, Kennedy’s approach^36^ was applied to obtain the maximum and minimum amounts of each input based on the ADQI. The pedigree matrix, the Kennedy’s approach coefficients, and the results of the variability of the Kennedy’s approach are found in Tables S3, S4, and S5, respectively, in the Supplementary Material. The final lower and upper bounds of AP2 are included in Table 2.

AP3 - This approach is slightly different from AP2. Although the input uncertainties are also derived from the pedigree matrix, they are based on the geometric standard deviation.^37^ Thus, for each input, a set of indicators in the form of standard deviation was selected, aggregated, and then converted to the final standard deviation of each input. Further details of this step can be found in Table S6 and Equation S1 in the Supplementary Material. The lower and upper bounds obtained for this approach are included in Table 2.

Step 2 consisted in defining the most appropriate distribution function for each approach and each input. In the case of probabilistic sampling, the distribution function plays a vital role. It is responsible for generating a random sample of the referred input. Thus, attention must be given to this step. Due to a lack of statistical data for the parameters under consideration, various distribution functions were tested to find the most appropriate for the model. This step of the methodology is limited to lognormal, normal, uniform, triangular, and PERT distribution functions, where their appropriateness with each input in all approaches was tested. The main requirement was the data necessary for running each sampling distribution function. For example, AP1 provides the upper and lower bounds and mean values of each input. The same is said for AP2. However, these bounds should not be taken as the variation bars of the data, instead, they refer to the maximum and minimum of that specific input. Unlikely, in the case of AP3, the standard deviation was the information obtained according to the approach. A function check sub-step was included to ensure a good fit of the distribution function relative to sampling limits and relative error to the reference case. After each sampling with a chosen distribution, the results were analyzed to check the consistency of their bound values. If the results and histogram were consistent, then they were approved, if not, a new distribution function was tested. For instance, the appearance of negative bounds suggested the inappropriateness of a certain distribution function. A negative flow could not be physically appropriate for the cases under investigation. An interactive Supplementary Excel File (input_sampling) is provided with instructions to check the distribution function. In this file, the default values are set to the input x_1_ for AP1, AP2, and AP3. Also, other researchers can benefit from these files for testing and implementing input distribution functions in their work.

Step 3 regards propagating the uncertainties to LCA model outputs (the indicators). For this, the ReCiPe 2026 (H) midpoint and end point indicators were used to generate the LCA constants of,^38^ obtained from Ecoinvent^39^ and SimaPro as software.^40^ Refer to eq 1 for the mathematical expression representing the LCA. At a midpoint level, many indicators require individual analysis. The end point indicators - human health, ecosystem quality, and resource availability- are most influenced by PMFP, GWP, TAP, and FFP, respectively. In the model represented by eq 1, x_i_ represents an input flow, Y_k_ represents the midpoint or end point indicator, a_i,k_ is the Ecoinvent factor for the corresponding input and indicator, and n represents the number of inputs. The factors (a_i,k_) for all inputs are presented in Table S7 in the Supplementary Material.

$$Y_{k}=\sum_{i=1}^{n}x_{i}a_{i,k}$$ 1

The use of this equation is justified by the assumption that each output (or indicator) is obtained from a linear equation, assuming that the inputs are not related to each other, which is a limitation in this study. In any case, this representation of the LCA model relates input and output in the form of y = f (x). For this study, let us consider the midpoint indicator GWP. It can be obtained by expanding eq 1 to eq 2, where n=8.

$$y_{\mathrm{GWP}}=x_{1}a_{1,\mathrm{GWP}}+x_{2}a_{2,\mathrm{GWP}}+x_{3}a_{3,\mathrm{GWP}}+x_{4}a_{4,\mathrm{GWP}}+x_{5}a_{5,\mathrm{GWP}}+x_{6}a_{6,\mathrm{GWP}}++x_{7}a_{7,\mathrm{GWP}}+x_{8}a_{8,\mathrm{GWP}}$$ 2

This same procedure is then applied to the remaining 17 midpoint indicators and 3 end point indicators of the ReCiPe method.

Step 4, LSA and GSA are used to verify how inputs contribute to outputs. GSA was carried out by calculating Sobol’s indices, which are based on the variance of the involved parameters. A sensitivity analysis library in Python (SAlib)^21^ was chosen to perform the GSA of the input-to-output parameters. SALib performs sensitivity analysis for any model that can be expressed in the form of f­(x)=y, such as eq 1.

For conducting LSA, authors may choose to consider the same variation for all input parameters, usually a small perturbation if following the matrix perturbation theory^41^ perturbing the data with a ± 1% variation^16^ or a ± 10% upper and lower bound. In this study, the upper and lower bounds of inputs after sampling were used. Thus, not all input parameters will vary in the same proportion. LSA relative indices ( S̲) are calculated to measure the influence of each input parameter on the outputs of the LCA model according to eq 1) of the literature review in the Supplementary Material.

Step 5 encompasses input importance based on the sensitivity analyses. The sensitivity indices from the previous step are useful to define the order of attention that stakeholders must consider in decision-making. Two categories for the sensitivity of the inputs are considered. Input sensitivity at a problem-oriented level refers to the sensitivity of inputs to the midpoint indicators. On the other hand, input sensitivity at a damage-oriented level relates to the sensitivity of inputs to end point indicators.

## 3 Results and Discussion

The results and discussion are presented in four subsections. The first deals with the uncertainty propagation throughout the model at a midpoint level, emphasizing the importance of using MC and contrasting the approaches employed. The second and third explain input sensitivity on the midpoint and end point levels using LSA and GSA. GSA and LSA are not compared due to their nature differing in application and interpretation, but both are used together for robust decision-making. The last one is dedicated to the practical implications of this work.

### 3.1 Uncertainty Propagation

For AP1, AP2, and AP3, it was found that PERT, triangular, and lognormal distributions fit best, respectively. AP1 and AP2 uncertainty was defined as upper and lower bounds, suitable for PERT and Triangular distributions, respectively. The function test showed good appropriateness of the results and errors compared to the reference case. For AP3, which had the standard deviation as a source of uncertainty information, the normal distribution could be reasonable, however, it displayed inconsistent results during the function test such as negative lower bounds. Physically, it is not acceptable as the flow of material cannot be negative, leaving the lognormal with consistent results, in accordance with the literature.^42^ The LCA mathematical expression is that of eq 1, exemplified in eq 2, to which the random input generated with MC is multiplied by its factor (for instance, x_1_a_1, GWP_) and then added up to the remaining inputs, for N repetitions. The outcome of uncertainty propagation through MC simulation for the approaches is shown in Figure 2. For simplification purposes, the inputs presented are bagasse and water, while the outputs are GWP and PMFP. The *x*-axis represents the amount of input sorted by range, while the *y*-axis represents the frequency at which these ranges appear in the total N runs that were carried out (N=10,000). All input parameters are sampled independently and then combined into the model. In the case of AP1, the PERT function fits best after propagating the uncertainty for all the inputs, and as only this function needed to be applied, the output distribution has the same tendency as that of the inputs. The same interpretation is valid for AP2 and AP3. This shows that the LCA model applied can combine the inputs’ uncertainties and propagate them to the outputs with the same distribution function.

**Figure 2:**
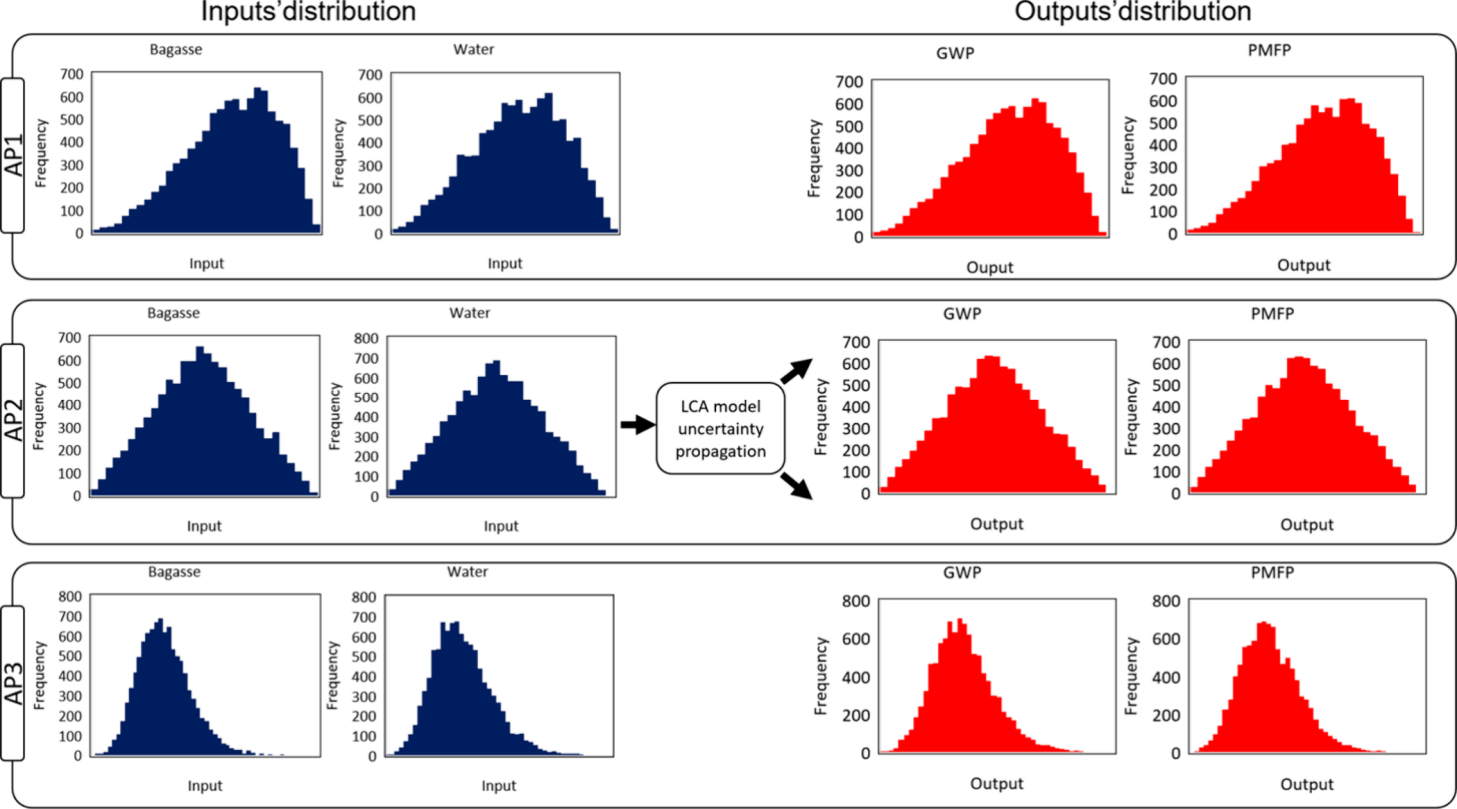
Uncertainties propagation from LCA inputs to outputs using MC with normal distribution and N=10,000. A confidence interval of 95% has been considered.

By sampling each input using MC, statistical information was obtained for the range of variation of the input. The main results are summarized in Table 3. The reference values represent the amount of each input without variation bars, i.e., the case in which no uncertainty would be included. In this study, the standard deviation (SD) indicates the spread of each data point within the sampling population. Sampling the inputs with MC also provided lower and upper bounds based on the sampling method and should not be confused with the limits initially attributed to the mean value. In this case, the MC sampling creates a random input that allows calculating a single output variable with eq 1 for a number N. Without the use of a sampling method, it would be impossible to generate N values for the output and define upper and lower bounds for both inputs and outputs. Obtaining the input bounds is fundamental as they are later used for GSA and LSA. Overall, for all parameters, the sampling replicates average values within acceptable ranges if compared with the initial values of the reference case. AP1 shows average values that deviate the least from the reference case in comparison with the other approaches. Even though this occurs, AP1 also shows the lowest SD, indicating a low variability in the data points. Emphasis should be given to the fact that the limits for AP2 obtained from the pedigree matrix were higher compared to those calculated for AP1. Another important note is that, as AP2 and AP3 have a more subjective nature, depending on the criteria that are applied to derive the DQI from the pedigree matrix, this scenario could change. This implies directly the variability of the data, meaning that parameters treated under AP1 experience a lower fluctuation, and consequently have lower maximum and minimum data points compared to AP1 and AP2.

**Table 3:** Statistical Analysis of the Foreground Inputs of the LCA Model for Both Approaches, with N=10,000 and a Confidence Interval of 95%^a^

Inputs	x_1_	x_2_	x_3_	x_4_	x_5_	x_6_	x_7_	x_8_
Reference	10.469	45.868	1.347	1.055	0.026	0.038	1.282	16.744
AP1 – after uncertainties propagation with MC								
Mean	10.213	45.088	1.349	1.055	0.025	0.037	1.252	16.346
SD	0.590	2.994	0.094	0.074	0.001	0.002	0.071	0.932
AP2 – after uncertainties propagation with MC								
Mean	10.448	45.761	1.341	1.054	0.026	0.038	1.284	16.768
SD	1.711	6.496	0.193	0.151	0.004	0.005	0.183	2.406
AP3 – after uncertainties propagation with MC								
Mean	10.683	46.656	1.375	1.076	0.027	0.039	1.307	17.051
SD	2.162	9.319	0.276	0.215	0.005	0.008	0.266	3.418

a SD – Standard deviation.

After independently sampling the inputs with MC, all the data points generated (10,000) were used in the LCA model, propagating the uncertainties to the LCIA midpoint indicators by means of eq 1. The results for environmental impact indicators are shown in Figure 3. Reference case (Ref) provides the absolute value of each environmental impact indicator, representing the case in which no uncertainty information is available, obtained from Simapro with Ecoinvent as the database after introducing foreground data. The objective is to show how the results obtained from AP1, AP2, and AP3 deviate from the reference. In this case, the interpretation of the results for the reference case is limited to a fixed value, however, it is known that due to certain aspects such as technology and geographical scenarios, these values vary to a certain extent. Unlikely, the results obtained using the proposed approaches AP1, AP2, and AP3 give a broader range of interpretation, allowing for accounting for unknown but yet expected variabilities in a real LCA study. This is one of the biggest advantages of MC for propagating uncertainties to the outputs (indicators). It allows the outputs to be reported along with statistical information. This means that in decision-making, the impacts must be analyzed from a perspective of possibilities, even though a mean value is provided, it should be considered that the indicator value is found in a range of possibilities with a maximum and a minimum. The minimum value would be ideal, and more results about this are presented with the sensitivity analysis in the next sections. Noticeably, for the indicators presented, the approaches propagated uncertainties and provided average values of indicators that deviate slightly from the reference case, with an error below 5%. For instance, the reference case GWP is 8.620 kg CO_2_ eq, while the AP1 was 8.443 kg CO_2_ eq It points to the good acceptability of the model uncertainty propagation, and the suitable choice of the distribution functions for the approaches. Details regarding the uncertainty propagation, output standard deviation, and relative error of the 18 indicators for AP1, AP2, and AP3 can be found in Tables S8, S9, and S10 of the Supplementary Material. Propagating uncertainty from inputs to outputs (environmental indicators) is important in informing the existence of fluctuations of LCA results, helping in decision-making.

**Figure 3:**
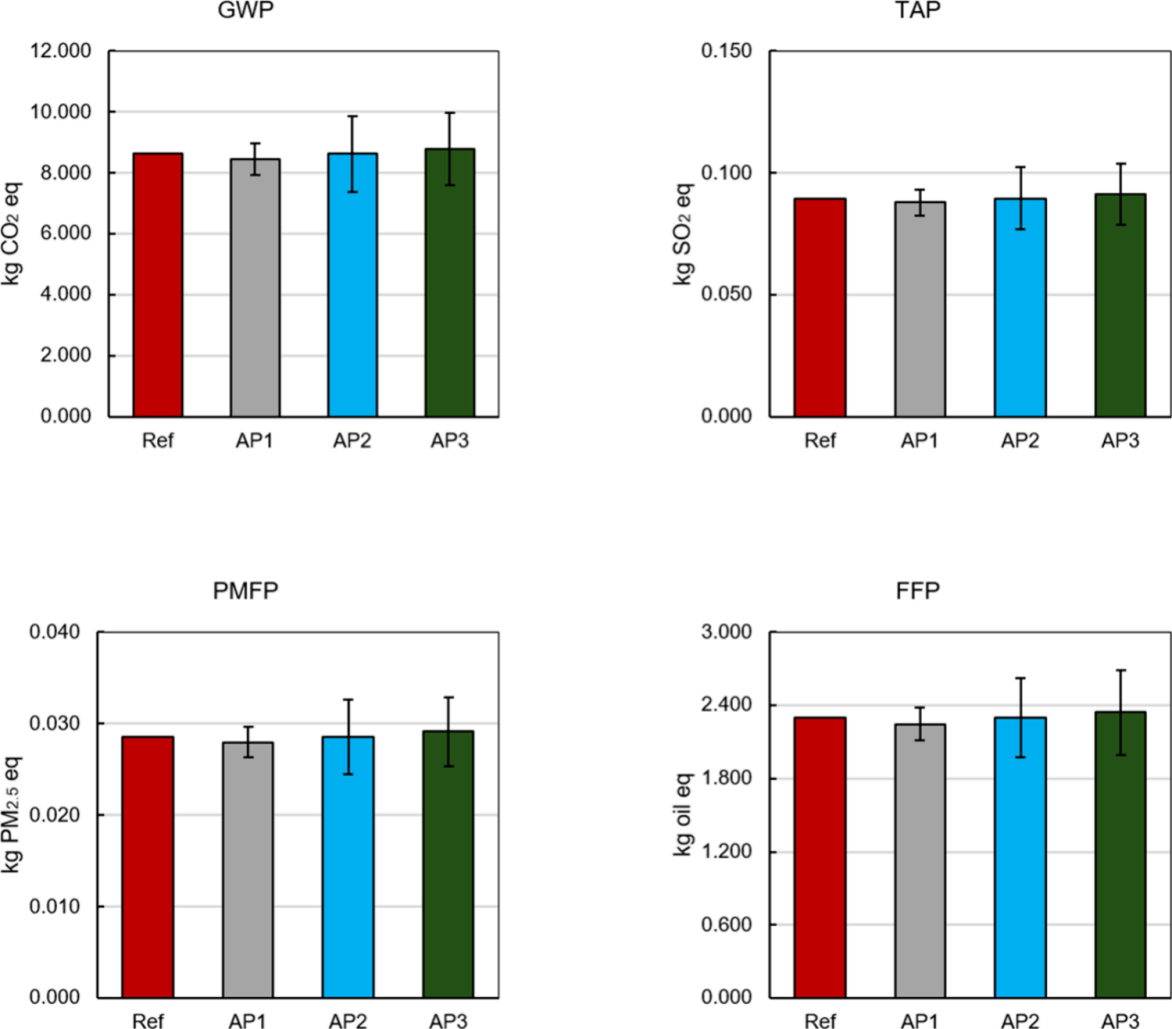
Midpoint indicators after uncertainty propagation for the three approaches and the reference case.

This work is, however, also useful to give insights into the uncertainty propagation in terms of damage areas, such as Human Health, Ecosystems, and Resources. The end point indicators are depicted in Figure 4 for Human Health (in DALY units), Ecosystem (in species.year), and Resources (in USD). The sampling was first propagated to each input individually (N=10,000), then to the midpoint indicators, finalizing with the application of the end point coefficients. This means that the uncertainty is cumulated and presented for the end point indicator. The error bars illustrate how uncertain an end point indicator is, corresponding to the variability in terms of lower and upper bounds. When comparing AP1, AP2, and AP3, slight differences among their average values are observed. Additionally, a tendency is observed in the behavior of the three approaches in contrast to the reference, in which the average slightly increases from AP1 to AP3. Looking at the results and error bars for Human Health, Ecosystem, and Resources, AP1 showed the lowest average value of the three approaches. For instance, the Human Health indicator using AP1 is 3.21 × 10^–5^ DALY, with lower and upper bounds of 2.60 × 10^–5^ DALY and 3.59 × 10^–5^ DALY, respectively. On the other hand, AP2 results in an average of 3.28 × 10^–5^ DALY, with lower and upper bounds of 2.12 × 10^–5^ DALY and 4.41 × 10^–5^ DALY, respectively. AP3 provided an average of 3.33 × 10^–5^ DALY with 2.32 × 10^–5^ DALY and 5.15 × 10^–5^ DALY as lower and upper bounds, respectively. AP1 also showed the lowest standard deviation, indicating it performs slightly better on average and with the least variability. AP3, on the other hand, performed with higher variability. It is important to emphasize that the results were propagated with different distribution functions as a result of having different statistical data for each input individually.

**Figure 4:**
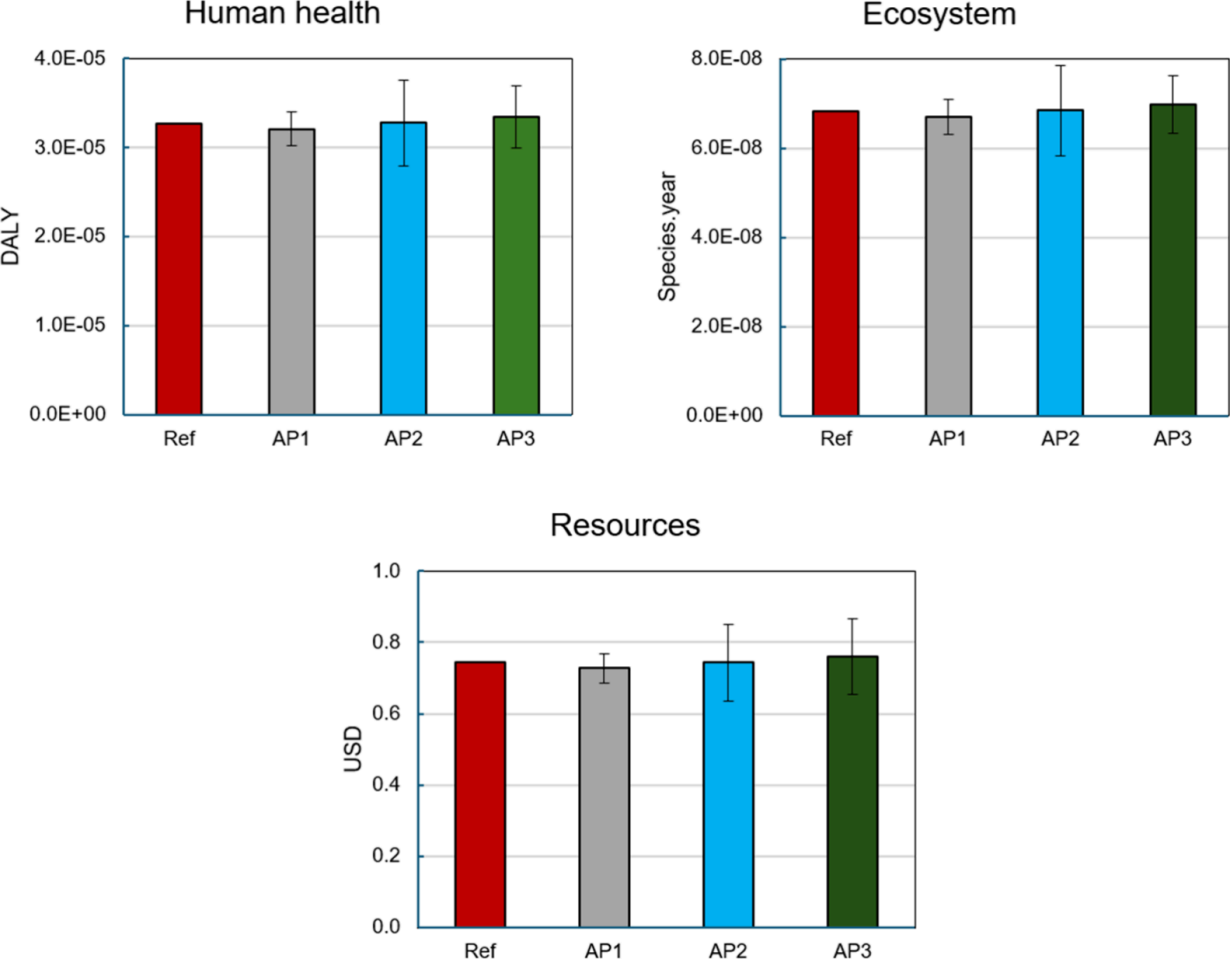
End point indicators after uncertainty propagation for the three approaches, using the ReCiPe 2016 (H) end point coefficients.

### 3.2 Input Sensitivity at a Problem-Oriented Level

After implementing the model in SALib for a GSA, the first-order Sobol indices were used to describe how the input uncertainties influence the variance of the outputs. Sobol indices vary from 0, which is the lowest contribution to uncertainty, to 1, which is the highest contribution. At a midpoint level, the first-order Sobol indices are shown in Figure 5. A general overview of the 18 indicators shows the variability of Sobol indices, suggesting that input contribution may differ according to the targeted midpoint indicator. In the GWP from AP1, input x_8_ (steam) has the highest index, meaning that its uncertainty contributes to more than 90% of the GWP uncertainties. This opens space for a more in-depth discussion, that the input with the highest material flow will not necessarily play the main role in the uncertainties. The inventory shows that the input x_8_ is three times lower than x_2_ (water), which has a higher range of uncertainty imposed by the upper and lower bounds, and yet, it influences the uncertainties of this indicator to a higher extent. A possible explanation is that the characterization factors, although considered constants, also play a role in increasing or decreasing the results of the model. The study revealed that for PMFP, input x_6_ (Raney-nickel) is responsible for nearly 86% of the uncertainties on the outputs, while the parameter that contributes the least is x_4_ (lime). The uncertainties in TAP were mainly impacted by the uncertainties related to x_6_ (Raney-nickel), and for the case of FFP, once again, steam was the key contributor to the uncertainties. The indices may vary slightly from the approaches for the same input and output. However, it was also found that for the same output (from the midpoint indicators list), independently of choosing AP1, AP2, and AP3, the first-order Sobol indices have similar behavior, and the order of input contribution is the same. This finding suggests that despite their differences and similarities, AP1, AP2, and AP3 are equally effective for conducting a GSA of the proposed case study. In all approaches, for some inputs and indicators, the Sobol indices appear as zero, however, they have extremely small contributions that are only observed when the index is rounded to four or more decimal digits.

**Figure 5:**
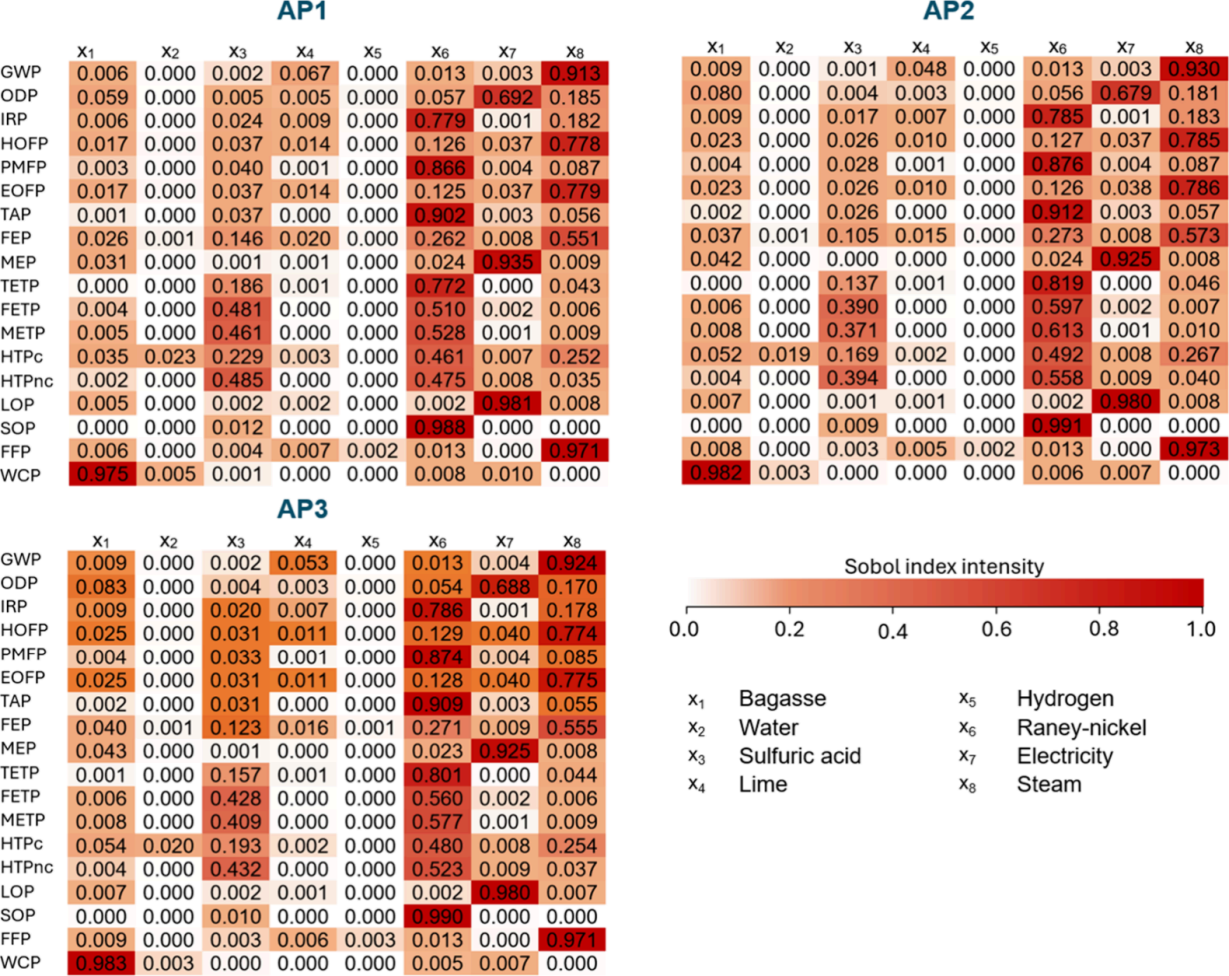
Overview of the first-order Sobol indices obtained with model implementation in SALib.

Evaluating the LSA using the range of variability differentiates this study from the traditional LSA, which normally considers only a fixed percentage variation on the input parameters. The procedure adopted in this paper uses the upper and lower values from the MC sampling, yet it is based on the one-at-a-time theory. An LSA analysis for AP1 is seen in Figure 6. Interestingly, the results reveal that the impacts were more sensitive to an increase than to a decrease in the input values, implying that increasing the input values causes more changes to the indicators than reducing the inputs. However, this is not an effect of the MC sampling only, but also accounts for the contribution from the database factor (a_i, k_), presented in Table S7. This can be seen as a consequence of the generated upper and lower bounds. An example is the bounds of x_8_, the parameter to which GWP is most sensitive, ranging from 13.25 to 18.14 kg, with a mean of 16.74 kg. Different from using a fixed percentage variation, in which a small percentage variation is imposed on the parameters, here the variations are based on the upper and lower bounds obtained for the inputs after MC sampling analysis, which means that not necessarily deviate the same from the mean. This can be corroborated by looking at the positive and negative variations of the outputs for each input (x_i_). Another important outcome from these results is that LSA shows the importance of inputs in line with the results of the GSA, as can be observed for GWP, PMFP, TAP, and FFP. An emphasis is given to the fact that both GSA and LSA have different meanings, although they report the most important inputs of an LCA model. In this way, the LSA is tied to the limits of the uncertainty propagation of the inputs, although not consider the effects of the uncertainty or variance.

**Figure 6:**
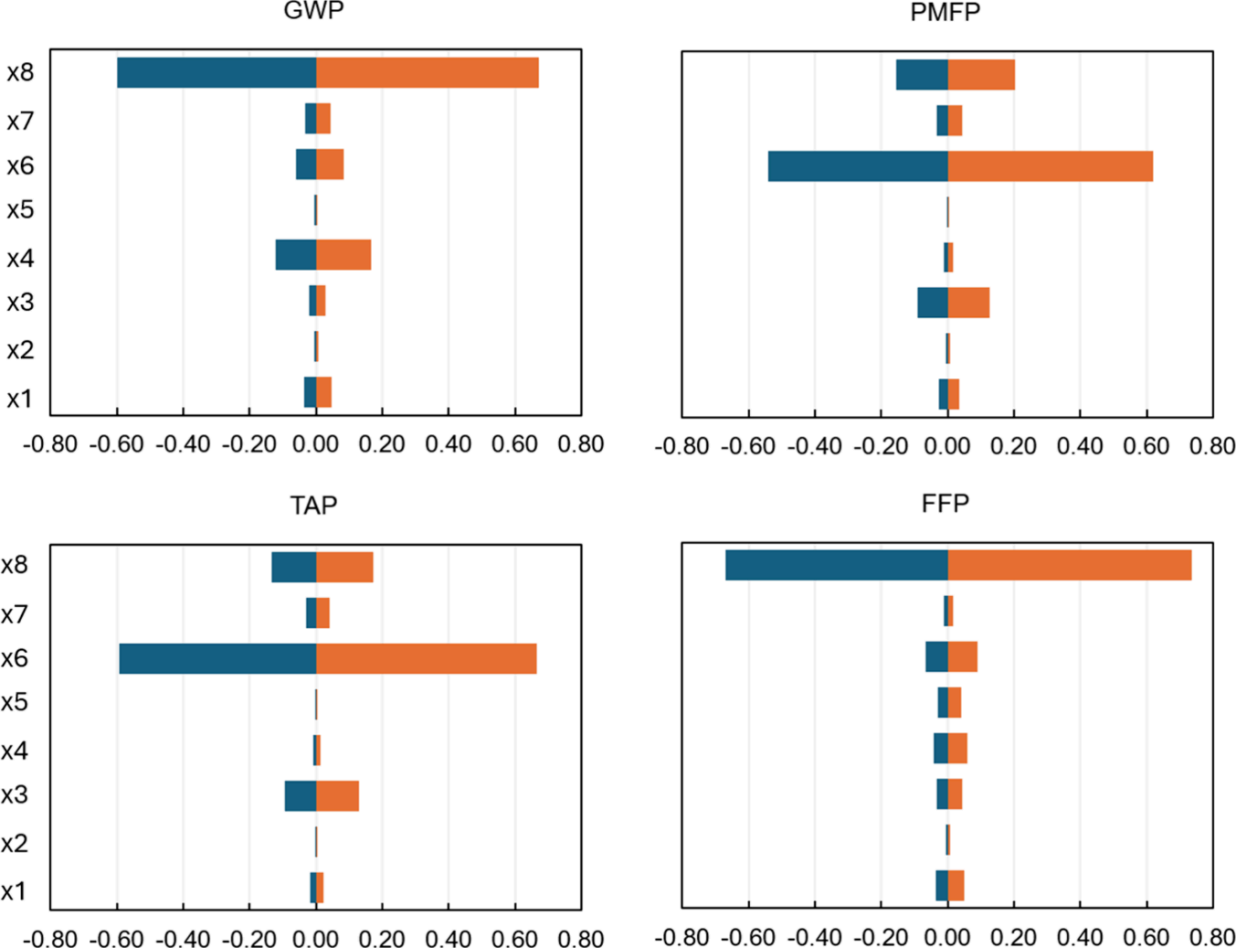
LSA indices for the 4 most important midpoint indicators using AP1.

In the sensitivity analysis, both LSA and GSA confirm that steam usage and nickel catalyst application are the primary contributors to most of the indicators. The results reveal similar patterns in the contribution of these inputs. Unlike GSA, which evaluates the impact of input variations across the entire parameter space on the output’s variance, LSA focuses on local sensitivities by examining the effects of perturbations within the upper and lower bounds of sampled values. This provides stakeholders with critical insights for identifying the most sensitive inputs to mitigate environmental impacts as determined by LCA results. For instance, maintaining the steam input close to its nominal value, rather than allowing it to increase towards the upper bound, can prevent a rise in GWP. Conversely, reducing the steam input towards the lower bound could lead to a beneficial decrease in GWP relative to the nominal output value. Although these two inputs are the most influential on a general basis, input sensitivity at a problem-oriented level requires more attention and the aim at indicators individually, which will depend upon the LCIA method utilized. In the case of the present paper, ReCiPe 2016 (H) delivers 18 indicators. Each indicator is sensitive to the 8 inputs of the case study and requires an analysis of how sensitive the indicator is to each input. More complexity arises if the number of inputs in the model increases. This can be simplified by using the proposed procedure for selecting the most important indicators, which will result in 4 indicators, i.e., GWP, PMFP, TAP, and FFP. It must be analyzed accordingly for each LCA model, consideration, and assumption.

### 3.3 Input Sensitivity at a Damage-Oriented Level

As an additional contribution to the field of LCA, this work also describes the relationship between inputs and damage areas in terms of global sensitivity analysis. This can be useful in investigating how input uncertainty could contribute to the final uncertainty of the end point indicators or damage areas, especially to make decisions aiming at reducing the impact on a damage-oriented level. To further describe the influence of the input on the output’s variance at a damage-oriented level, a GSA of the three ReCiPe 2016 (H) end point indicators was conducted. The results of the Sobol indices are presented in Figure 7. Looking individually at each damage area, the indices obtained by applying AP1 show that, in comparison to AP2 and AP3, the input sensitivities are very similar, except for x_1_ and x_8,_ which differ for the three approaches in Ecosystems. This is evidence that the approaches are performing accordingly. Different from the problem-oriented level, it is observed that at a damage-oriented level, more inputs stand out with a higher sensitivity index. For instance, the GSA for Human health with AP1 shows that the most sensitive parameter is x_6_, with an index of nearly 60%, however, x_8_ also has a degree of importance, with an index of nearly 30%, followed by x_3_ and x_1_ with minor contributions. The same behavior is seen for the Ecosystem, with a smaller difference among the most sensitive indices (x_8_, x_6_, x_1_). Unlikely, at a problem-oriented level, the GSA shows that x_8_ is the most sensitive parameter for GWP, with more than 90% of the contribution, as a comparison. The results show that at a damage-oriented level, the complexity of analyzing the GSA indices is dramatically reduced as it requires aiming at 3 indicators only, which means more simplicity and objectivity when evaluating the sensitivity of parameters and making decisions. Nevertheless, the mathematical relationship between midpoint and end point indicators and their implications should be considered. As an end point indicator is derived from the combination of midpoint indicators, it results that a reduction in environmental impacts at a damage-oriented level also implies on reduction in the midpoint indicators. For instance, if decisions should be made based on the Ecosystem indicator by acting on the most sensitive inputs, x_8_, x_6,_ and x_1_, at a problem-oriented level, the WCP, FEP, FETP, ODP, TEP, TAP, and LOP indicators would be affected automatically, but at different proportions since their contribution to the Ecosystem damage differs. The control of the changes would remain at a damage-oriented level, that is, at the end point indicator Ecosystem. It suggests that aiming at input importance at the damage-oriented level reduces complexity and benefits changes at the midpoint indicators.

**Figure 7:**
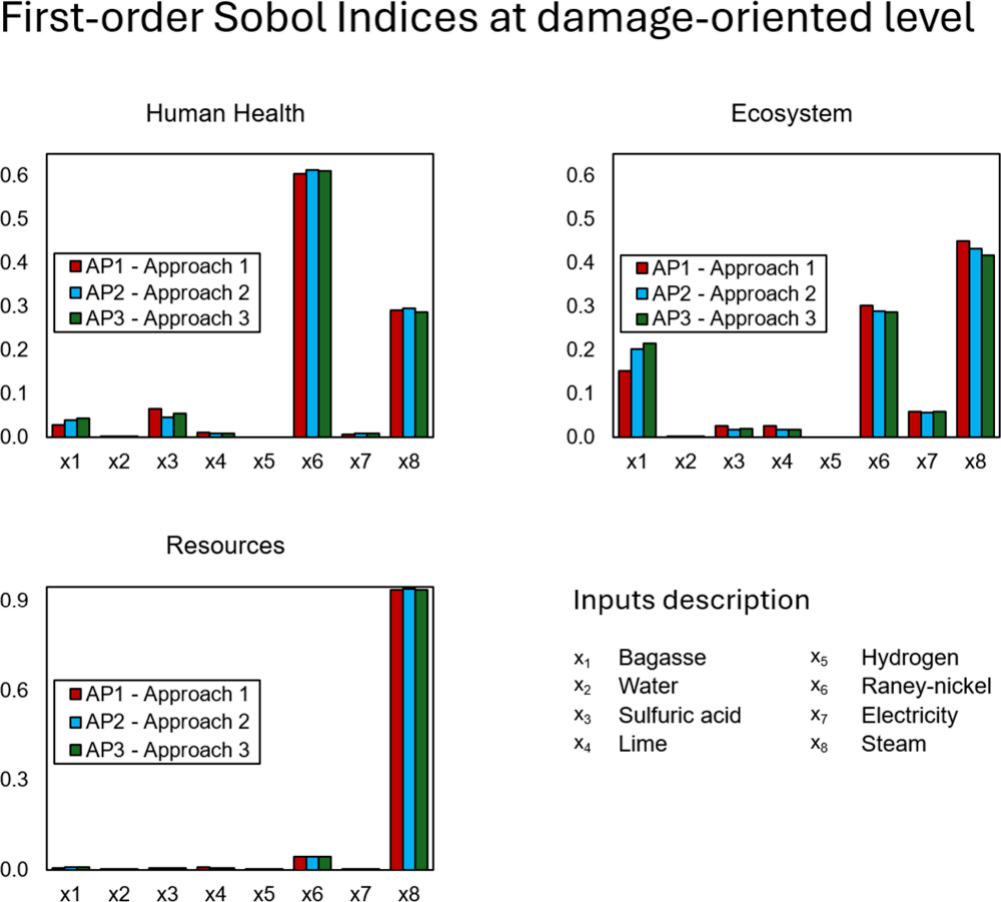
Sobol indices for input sensitivity at a damage-oriented level.

The end point indicators are especially relevant in health and ecosystem conservation policies. Once quantifying the potential damage to the areas of human health, ecosystem, and resources, the results can lead to decisions towards mitigating negative impacts. The end point indicator to human health, for instance, is affected by problems like an increase in respiratory disease, an increase in cancer, or even an increase in malnutrition.^38^ Meanwhile, the indicator damage to ecosystems takes into account damage to freshwater species, to terrestrial species, and to marine species.^38^ By propagating uncertainty to these indicators, their effects from the LCA point of view are otherwise evaluated as a broader range of possibilities instead of punctual values, which can be useful in setting policies aiming to minimize the impacts in the production of PCMs. Moreover, the sensitivity analysis, either LSA or GSA, is proven to be useful when setting targets on the most sensitive input, relating them to potential changes in production pathways and promoting policies to cleaner practices at the production level of such components.

### 3.4 Final Remarks

The practical implications of such a work reflect the objectiveness of defining quantitative uncertainty, opening ways to be extended to other bio-based PCMs. In addition, the general framework can also be applied to other LCA studies to both propagate uncertainty and calculate input sensitivity. AP1 could serve as an additional approach for defining parameter uncertainties, complementing, not substituting, conventional approaches such as the use of a pedigree matrix, used in AP2 and AP3. Following the uncertainty definition, the structured methodology is presented, proposing a procedure for uncertainty propagation, and the degree of importance of inputs based on GSA and LSA. Also, an easy-to-use procedure for identifying the most sensitive inputs at a problem-oriented and damage-oriented level is developed and can be extended to other LCA studies, regardless of the field of study.

Despite the importance of data uncertainty in LCA, it has been evidenced that only a few studies are dedicated to it, and that this issue expands across many other research areas, such as thermal energy storage. To contribute to this topic, this paper proposed a novel 5-step methodology for treating data uncertainty, uncertainty propagation, sensitivity analysis, and input sensitivity. The investigated literature concentrated on the sensitivity of inputs at problem-oriented, reporting their importance on the midpoint indicators. In this work, the discussion of input sensitivity was extended to a damage-oriented level, discussing input importance based on the end point indicators. Within its scope, an additional quantitative approach for determining parameter uncertainty was proposed and tested with an application to bio-based PMC. GSA and LSA were conducted to compare the proposed approach with conventional ones.

The proposed approach (AP1) performed similarly to the conventional qualitative approaches (AP2 and AP3). After MC sampling, AP1 delivered indicators in good agreement with the case where no uncertainty is known. Moreover, the Sobol indices obtained for AP1 indicated the same order of contributions to output variance as for AP2 and AP3, meaning it can identify the same sensitive parameters for the LCA model at mid and end point levels. Attention is given to the fact that this approach is primarily tested for the foreground input parameters and that a higher uncertainty is expected at the end point indicators due to the mathematical manipulation of data.

LSA conducted with the lower and upper bounds from MC sampling was revealed to be useful. Its application identified the same sensitive inputs as in the GSA. However, it requires a different interpretation as the latter provides valuable information regarding input contribution to output variance, while the former gives information on output changes relative to input variation. LSA performed with lower and upper bounds from MC sampling is thus recommended.

The use of AP1 pointed to the same sensitive indicators as AP2 and AP3, for either GSA or LSA. At a problem-oriented level, defining input sensitivity needs investigation around each indicator individually. As the methodology proposes, this can be simplified by selecting the most influential indicators, which overall shows that attention should be given to steam and Raney-nickel. On the other hand, input sensitivity at the damage-oriented level already reduces the number of indicators, requiring an analysis of more than one input, but impacting directly on the midpoint indicators.

The proposed five-step sensitivity analysis methodology, although initially developed for lignocellulosic biomass or bio-based PCMs, can be adapted to other fields. For example, in battery production, input parameters such as energy mix, metal extraction yields, or recycling rates can be assigned uncertainty bands and probability distributions. In building insulation, material density, thermal conductivity, and service life can be similarly assessed. For bioplastics, feedstock type, fermentation efficiency, and polymerization rates can serve as uncertain inputs. In each case, starting from an expression that represents the LCA model, the same structured approach, such as defining uncertainty, selecting probability distributions, propagating uncertainty, conducting sensitivity analyses, and insights on the midpoint or end point level can be applied to assess the robustness and influence of key LCA parameters across diverse applications.

The main advantage of the methodology used in this study is to propagate uncertainties from foreground data. That is, the LCA practitioner often does not have background data to form a complete inventory of the process, resorting to the database, where only foreground data is needed, which is the data that is constantly obtained as a multiplication factor by the indicator value already calculated through the database. One of the limiting factors in this study refers to the aggregated value of the uncertainty of the database coefficient, treated in the study as parameter a, which is not always available in the database. Studies could also concentrate attention on defining the relationship among parameters, considering nonlinearities in the mathematical correlations for the uncertainty propagation and sensitivity analysis. Considering the relationship between material input and output flows, material stock and residues re-use as fuels, for example, could help define a nonlinear expression for the LCA model. Its inclusion in future studies could enhance the results of indicator uncertainties, making them more robust.

## Supplementary Material





## Data Availability

We declare that all data supporting and generated in this study are available as stated within the text, as well as in the Supplementary Material

## Notes

Literature review, methodological procedure, case study boundary flow, material flow, calculations of parameter uncertainty, Pedigree matrix, Kennedy's approach, results of the variation, coefficients used, Ecoinvent factors, and uncertainty propagation (PDF)
Input sampling: Tool for sampling with Monte Carlo (XLSX)
