# Supplementary material for: Uncertainty Propagation and Input Sensitivity in Life Cycle Assessment: An Application to Phase Change Materials
Source: ACS Sustain Resour Manag. 2025 Aug 18;2(8):1593–604. doi: 10.1021/acssusresmgt.5c00298 (PMC12403139; doi:10.1021/acssusresmgt.5c00298)
Supplement: Supplementary file 1 [file rm5c00298_si_001.pdf]

Uncertainty propagation and input sensitivity in life cycle assessment: an application to phase change materials

Humberto Santos[a*], Silvia Guillen-Lambea[a]

[a]Aragón Institute for Engineering Research (I3A), Thermal Engineering and Energy Systems Group, University of Zaragoza, Agustín de Betancourt Building, C/María de Luna 3, 50018, Zaragoza, Spain

*Email: hdasilva@unizar.es

Literature Review

Data quality

Data quality has been defined in ISO 14040/14044 as the aspects of data that relate to its ability to satisfy established requirements, related to the goal and scope of the LCA under study (ISO 2006a, b). In this respect, the US Environmental Protection Agency has developed a guide for data quality indicators (DQI) using the pedigree matrix (Edelen and Ingwersen 2016). Using the pedigree matrix, an indication of uncertainty can be obtained without measured variability information (Qin et al. 2020). This approach utilizes five qualitative indicators to determine scores that represent data uncertainty, varying from 1 to 5 (Weidema and Suhr 1996). The indicators used are source reliability, completeness, temporal correlation, geographical correlation, and technological correlation. Over the years, the application of this methodology has been improved and is used, for instance, by Ecoinvent to implement the geometric standard deviation in their unit processes (Frischknecht et al. 2005; Ciroth et al. 2016).

In LCA, data uncertainty can be divided into three categories: model, scenario, and parameter uncertainty (Lloyd and Ries 2007; Bamber et al. 2020). Model uncertainty is related to the structure and mathematical relations of the model. On the other hand, scenario uncertainty is related to normative choices, such as functional units, time horizons, and geographical scales, for instance. Parameter uncertainty regards the inherent variability and randomness of values used in the LCA model, due to the difficulty in measuring exact values or even the lack of accurate values. According to Heijungs and LenzenHeijungs and LenzenHeijungs and Lenzen, uncertainty shows up in LCA in different manners such as input data, procedure assumptions, and data error propagation (Heijungs and Lenzen 2014).

Monte Carlo (MC) analysis has been used in different studies to understand how uncertainties propagate in LCA models (Sun and Ertz 2020; Bacatelo et al. 2024). In addition, most LCA software, such as SimaPro, Gabi, Umberto, OpenLCA, CMLCA, and Brightway 2, apply MC for parameter uncertainty propagation from inputs to outputs (Igos et al. 2019). As uncertainty is present in LCA, during the model realization, these uncertainties add up and affect the results, for which Monte Carlo analysis helps verify how the combination of the inputs influences the model's outputs (Golsteijn 2015). The most utilized distribution functions in this context are the normal, triangular, uniform, and lognormal probability functions (Lloyd and Ries 2007). If possible, the probability function can be derived based on data observation, however, this practice is not always possible. For instance, Ross et al. for their study, derived the distribution function of power consumption based on the observation of data variation amongst 107 residences in each month (Ross and Cheah 2019). Additionally, comparing distinct distribution functions, they show that the choice does not necessarily affect the mean GHG, still, the uncertainty can differ, especially from the triangular distribution compared to normal and lognormal distributions.

Sensitivity analysis methods

Sensitivity analysis is widely used to assess input uncertainty in LCA models, with global (GSA) and local (LSA) approaches being common (Wei et al. 2015). A key tool for GSA is the Python-based SALib library, which provides various sampling and analysis methods (Herman and Usher 2017). However, its application remains limited. On polygeneration systems, SALib analyzed scale-up impacts and economic uncertainties like fuel costs based on engine type (Schröder et al. 2019). Recently, Stajić et al. (2024) used it natural gas prices variation in a global international market based on Sobol indices, showing crude oil as the most influential factor (Stajić et al. 2024). In building energy performance optimization, SALib helped assess key parameters affecting heating and cooling in Moroccan buildings (Serbouti et al. 2018).

Sensitivity analysis is an important step when considering the data quality of an LCA study. There exist mainly three types of sensitivity analysis (Groen et al. 2014): the first is local sensitivity analysis (LSA) which can be conducted using the one-factor-at-a-time approach (OAT) or the matrix perturbation theory (MP); the second is the screening, conducted through the method of elementary effect (MEE); third is the global sensitivity analysis (GSA) carried out with a few different methods, such as standardized regression coefficients (SRC); key issue analysis (KIA), random balance design (RBD) and Sobol's indices (SME and STE).

LSA and GSA will be mainly discussed in this study. A few differences can be remarked: LSA can become easier because it requires only point values (central values), while GSA needs central values and parameters of dispersion; in terms of results, LSA gives ranking of sensitive input parameters and GSA results in uncertainty distribution of output(Groen et al. 2017). Wei et al. compared both LSA and GSA for LCA models, concluding that LSA is well adapted for a high number of uncertain parameters and that it is useful for finding the main driving factors of a model, while GSA methods determine the uncertainty over the domain of variation of parameters, including interaction among parameters (Wei et al. 2015).

LSA is commonly the most used type of sensitivity analysis due to its simplicity. In this analysis, inventory inputs are varied independently to investigate the influence on the LCA outputs. This means that while one input parameter receives a small perturbation, the others remain constant, and the influence on the environmental impacts is visualized (Wei et al. 2015). The main limitation is that it does not consider the correlation between parameters, and they must be analyzed one-at-a-time (Zhang et al. 2015). Still within LSA, the other approach would be the matrix perturbation (MP) theory (Heijungs and Suh 2002). This theory considers mainly two types of flow associated with the LCA model: (i) the economic flow, which includes inputs and outputs of materials, products, services, energy, and waste; (ii) the environmental flow, which consists of materials extracted directly from the environment as wells as emissions from the unit processes to the environment. The perturbation on the elements of the matrices and the resulting influences will be directly reflected in the outputs. A mathematical expression for the LSA is represented by Equation 1.

$$\underline{S}(X_i, p_j) = \frac{\Delta X_i / X_i}{\Delta Y_i / Y_i} \tag{1}$$

Where ΔX_i represents the variation of input X_i and Δy_i the corresponding changes to output Y_i.

On the other hand, there is the GSA, which considers the effects of uncertain factors. It is defined as the study of how output uncertainty can be apportioned to different uncertainties in the model input (Saltelli et al. 2004). GSA can be independent, meaning that the correlations among input variables are neglected, or dependent, when the correlation between two or more variables is included (Wei et al. 2015). Moreover, GSA provides a detailed examination of the behavior of a variable, allowing multiple input parameters to be varied simultaneously (Al et al. 2019). GSA can be carried out in 5 steps (Saltelli et al. 1999; Groen et al. 2017). In step 1, input parameters and their uncertainties are distributed using probability density functions. Then, the uncertainty needs to be propagated within the model, which consists in step 2, and this is normally done by using Monte Carlo (MC) simulation. After the MC is concluded, the variance of the outputs is obtained (step 3). Step 4 consists of selecting a feasible method for calculating the sensitivity analysis, and finally, step 5 is the determination of the contribution of each input parameter to the outputs.

There are distinct approaches for conducting GSA based on different criteria (Reed et al. 2022). Some are derivative-based, elementary effect, regression-based, and variance-based methods. Within the variance-based approaches, Sobol is a classical method for computing sensitivity analysis delivering the so-called Sobol indices (Sobol 2001). S_i is the first-order Sobol indices and provides information on individual effects, and the total order Sobol indices (S_{Ti}) provide the effects on the outputs considering interactions between input variables. The first-order indices indicate the individual contribution of inputs, not considering interactions that may exist among the variables (Al et al. 2019). Its mathematical formulation is represented by Equation 2.

$$S_i = \frac{V[E(Y|X_i)]}{V(Y)} \quad (2)$$

V denotes variance, E is the expectation, and X_i the input variable being treated.

The total sensitivity analysis index (S_{Ti}) quantifies the influence of input on model outputs, considering all its interactions with other inputs. Mathematically, S_{Ti} is calculated by Equation 3.

$$S_{Ti} = 1 - \frac{V[E(Y|X_i)]}{V(Y)} \quad (3)$$

Approaches and methodologies previously developed

Various methodologies for uncertainty and sensitivity analysis are extensively documented in the scientific literature. Geisler et al. developed a methodological approach for uncertainty analysis in LCA (Geisler 2003; Geisler et al. 2004, 2005). They employed a series of equations grounded in process performance to estimate the maximum and minimum possible values for each flow, representing the most favorable and most unfavorable scenarios, respectively. They used data obtained from industrial facilities, supplemented by insights from experts in the chemical industry. Furthermore, dispersion factors were established for the elementary flows based on inventory analysis and production process efficiency, enabling the characterization of extreme scenarios for the CML baseline method indicators.

In a different study, uncertainty propagation and sensitivity analysis within the inventory phase of LCA were carried out (Wei et al. 2015). The researchers employed a matrix-based LCA approach incorporating correlations between input parameters and using uncertainty data derived from Ecoinvent 2.1 (2009). The methodology was tested using a case study involving a glass wool blanket, commonly used in construction, with a functional unit defined as 1000 kg of glass wool blanket. Apart from the midpoint indicators, the authors also used endpoint indicators in the sensitivity analysis. They used IMPACT+2002 for assessing the environmental indicators. Their study concluded with a proposed framework to guide the selection of appropriate sensitivity analysis approaches based on the available uncertainty information and specific analytical requirements. This methodology helps to determine the most appropriate sensitivity analysis technique tailored to individual cases, improving the robustness of LCA results.

Another study shows the use of variance-based and distribution-based methods to develop a global sensitivity analysis protocol testing a noise model to quantify the impact on humans exposed to noise from various sources during their life cycle (Cucurachi et al. 2016). Their protocol model consists of identifying uncertain input parameters and their relationship with the output parameters, followed by the propagation of uncertainties and subsequently the global sensitivity analysis using the R software (https://www.r-project.org/). The proposed methodology is robust and allows its extension to various life cycle assessment sensitivity studies. (Di Lullo et al. 2020) developed a general framework to enable the communication of parameter importance in LCA, using Morris and Sobol's methods for sensitivity analysis. Interestingly, they use a bottom-up engineering fundamental LCA model, starting with screening and sensitivity, and finalizing with an approximation of the initial model. This work seems to be very useful, especially for models with a high number of parameters. Additionally, (Qiao et al. 2025) proposed a stochastic framework for the comparative assessment of carbon emissions, energy consumption, and acidification potential. The application of this approach involves a multi-step process, starting with a sensitivity analysis to identify and quantify the most influential parameters. This is followed by the implementation of a pedigree matrix to characterize and define input uncertainties. Finally, a Monte Carlo simulation is employed to propagate these uncertainties, allowing a probabilistic assessment of environmental impacts. Although initially applied to a specific case study involving asphalt paving systems, this methodology has the potential to be adapted and extended to various LCA contexts in different sectors.

To improve the environmental assessment of biorefineries, (Shi and Guest 2020) developed the BioSTEAM, an open-source life cycle assessment (LCA) platform implemented in Python. Initially, the platform was applied to assess ethanol production from sugarcane. The approach incorporates uncertainty analysis for material flows, using inventory uncertainties, data quality (through the Pedigree matrix), and uncertainties in design and operational parameters. This allows sensitivity analysis through Spearman's

rank-order correlation. The framework facilitates rapid sensitivity assessments, with the ability to extend the analysis to other unit processes. A significant advantage of BioSTEAM is its compatibility with several life cycle impact assessment (LCA) methods, including TRACI, ReCiPe, IPCC, Impact2002+, and Usetox, among others.

Based on the literature review, it has been identified that research has concentrated its focus on the LCI phase. This means that the LCA practitioner needs to handle the LCI emission factors directly. In most studies nowadays, software integrated with a database such as Ecoinvent is used, where inputs are provided in the form of foreground data. Additionally, the published works tend to concentrate attention on a problem-oriented indicator, or midpoint indicator, such as global warming potential or another indicator individually. Therefore, the dynamics between uncertainty from the midpoint to the endpoint and the sensitivity to inputs are not discussed in detail. Thus, this work considers the ReCiPe 2016 midpoint (H) and endpoint to provide further insights into this discussion. The methodological framework is supported by the use of 5 steps through which LCA practitioners will be allowed to identify foreground parameter uncertainty, select the best distribution function, and propagate uncertainty from input to LCA outputs, finally quantifying the contribution of inputs through GSA and LSA. In the first step, an approach for quantifying uncertainty based on the cellulose content. Furthermore, attention is concentrated on the LCIA phase, instead of treating the data during the inventory phase. This is especially helpful for practitioners using databases like Ecoinvent, and various LCIA methods can be used. Input sensitivity is discussed not only in the midpoint indicators. This work extends the discussion to the endpoint level to understand what the order of inputs' importance is, since an endpoint indicator is the combination of midpoint indicators according to the factors of the method used in the LCIA phase.

Methodological procedure

S1. Life cycle assessment

The LCA boundaries of the case study considered are depicted in Figure S1.

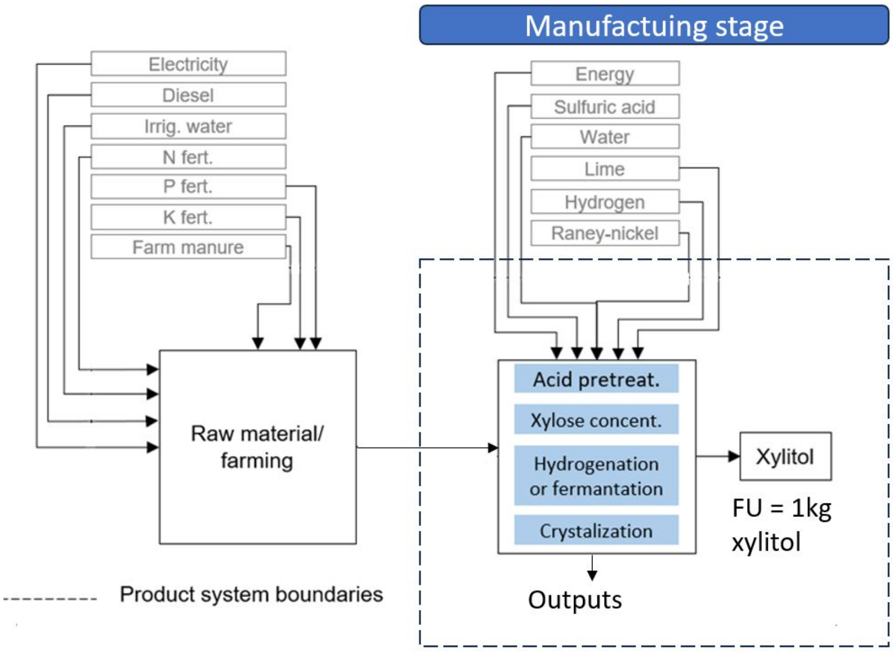

Figure S1. Case study boundary flows.

In this life cycle analysis (LCA), the chemical production pathways for xylitol from sugarcane bagasse is considered. The final objective is to carry out an environmental assessment of the production of 1 kg of xylitol, which is the functional unit (FU) of this LCA. It should be noted that sugarcane bagasse includes

all the impacts associated with the cultivation process, as considered in this study. The approach adopted follows the LCA guidelines established by the International Organization for Standardization (ISO) (ISO 2006a, b), where LCA is subdivided into four stages: (I) definition of objectives and scope, (II) life cycle inventory (LCI) analysis, (III) life cycle impact assessment (LCIA), and (IV) interpretation. These stages are discussed in detail below.

(I) Definition of objective and scope: The objective is to quantify the environmental impacts associated with xylitol production through a life cycle analysis. The scope is limited to a cradle-to-gate assessment of the plant, covering the manufacturing phase. The study boundaries include the cultivation stages and the stages involved in the manufacturing process, but exclude the equipment and facilities used in production. Specific details of the boundaries are presented in Figure S1.

(II) Inventory analysis: the life cycle inventory (LCI) considers the inputs and outputs flow to the production of xylitol, in the form of energy or mass, in the manufacturing stage. For example, it was considered the inputs to obtain the biomass feedstock during farming, electricity, steam, H_2SO_4, and etc. The inventory, regards the chemical production of xylitol, also from sugarcane bagasse (Karka et al. 2017; Özüdoğru 2018). For the geographical scenario, it was maintained with in the Indian scenario, and so all input processes from Ecoinvent were prioritized to be within this region, but whenever an input was not available, global {GLO} or rest of the world {RoW} markets were selected. The detailed information about the inventory inputs are included in Tables S1.

(III) Impact assessment: The life cycle assessment (LCA) focused on converting emissions associated with inputs and outputs into environmental impacts. To do so, each of the inputs and outputs of the unit process (UPR) were defined, assigning their corresponding emissions using the Ecoinvent database used in this study. These emissions were then translated into impact categories using characterization factors provided by the selected method, ReCiPe Midpoint (H) 2016. The Midpoint characterization factors were chosen to provide a perspective oriented to the specific environmental problems of xylitol production, allowing the identification of 18 impact categories for the assessment. To simplify the analysis, the LCA was performed using SimaPro software.

(IV) Interpretation: This stage focuses on the analysis and discussion of the results obtained in the different impact categories, as well as on the identification of the main contributors to these impacts. However, this phase is not necessarily the last one, as it can be carried out in parallel with the other phases.

Table S1. Material flow for xylitol production

**Chemical production of xylitol**				**Ref.**
Farming				(Shaji et al. 2022)
Inputs		Outputs		
Electricity (kWh)	**0.018**	Bagasse (kg)	9.145	
Diesel (kg)	**0.003**	Nitrogen oxides (kg)	0.004	
Water (kg)	**659.000**	Phosphorous runoff (kg)	0.001	
Nitrogen (kg)	**0.007**	NHS emissions (kg)	0.0006	
Phosphorous (kg)	**0.002**			
Potassium (kg)	**0.002**			
Farm yard manure (kg)	**0.241**			
Pesticides (kg)				
Manufacturing				(Karka et al. 2017; Özüdoğru 2018)
**Inputs**		**Outputs**		
Materials				
Bagasse (kg)	**10.469**	Xylitol (kg)	1	
Water (kg)	**45.868**	Waste inhibitors (kg)	6.563	

Sulfuric acid (kg)	**1.347**	Evap water (kg)	5.850
Lime (kg)	**1.055**	Glucose-rich (kg)	20.845
Hydrogen gas (kg)	0.026	Lig + cell + others (kg)	6.278
Raney-Nickel (kg)	0.038	Losses (kg)	0.116
		Waste water (kg)	10.282
		Gypsum (kg)	2.566
Energy			
Electricity (kWh)	**1.282**		
Steam (kg)	**16.744**		

S2. Data uncertainty

Table S2. Calculation of parameter uncertainty for AP1.

Input	Explanation	Values obtained	Reference
Water (kg)	Its variation is based on the amount of water for the dilution of sulfuric acid.	According to the amount of used and the ratio with water, this gives a variation of 35.56-51.68.	
H_2SO_4 (kg)	The amount of acid obeys the mathematical relation $m_{H2SO4} = 0.45m_{hemi}$	That gives 0.11 kg to 0.16 kg of H_2SO_4 for the range of hemicellulose content in sugarcane bagasse (25-30%). This result is nearly 1.1 to 1.6 kg since approximately 10 kg of H_2SO_4 is needed for each kilogram of xylitol.	(Fatehi et al. 2014)
Lime (kg)	The amount of lime (CaO) is provided at based on the chemical reaction $H_2SO_4 + CaO \rightarrow CaSO_4 + H_2O$ $(100\%\ conversion\ assumed\ for\ H_2SO_4)$	The ratio of CaO_2 was 0.784 kg CaO_2 per kg of H_2SO_4. This results in a range of 0.86 to 1.25 kg of CaO_2	(Özüdoğru 2018)
Raney nickel (kg)	Renay-nickel can vary in the amount of 3-30% relative to the xylose content, which in the best condition can be 26.7% (266.73 mg.g-1)	By calculating the range for Raney-nickel (25% hemicellulose) it gives a range of 0.02-0.2 kg per kg of xylitol. Xylose production from sugarcane bagasse by surface response methodology	(Paiva et al. 2009)
Hydrogen (kg), electricity (kWh), and steam (kg)	As clear relationships were not found for calculating these parameters individually, they were considered as a proportion based on the current inventory and the amount of xylose present on the feedstock.		

Table S3. Pedigree matrix (Weidema and Suhr 1996).

Indicator scores	1	2	3	4	5
Indicators, which are independent of the study in which the data are applied					
Reliability	Verified data based on measurements	Verified data partly based on assumptions on non-verified data based on measurements	Non-verified data partly based on assumptions	Qualified estimate (e.g. by an industrial expert)	Non-qualified estimate or unknown origin
Completeness	Representative data from a sufficient sample of sites over an adequate period to even out normal fluctuations	Representative data from a smaller number of sites but for adequate periods	Representative data from an adequate number of sites but for shorter periods	Representative data from a smaller number of sites and shorter periods, or incomplete data from an adequate number of sites and periods	Representativeness unknown of incomplete data from a smaller number of sites and/or from shorter periods
Indicators relating to the technological and natural production conditions under which the data are valid, and therefore dependent of the data quality goals for the study in which the data re applied					
Temporal correlation	Less than 3 years of difference to year of study	Less than 6 years of difference to year of study	Less than 10 years of difference to year of study	Less than 15 years of difference to year of study	Age unknown or more than 15 years of difference to year of study
Geographical correlation	Data from area under study	Average data from larger area in which the area under study is included	Data from area with similar production conditions	Data from area with slightly similar production conditions	Data from an unknown area or with very different production conditions
Further technological correlation	Data from enterprises, processes and materials under study	Data form processes and materials under study but from different enterprises	Data from processes and materials under study but from different technology	Data on related processes and materials but from same technology	Unknown technology or data on related processes or materials but from different technology

Following the indication of scores in the pedigree matrix, the Kennedy (Kennedy et al. 1996) method was applied as per Table S4.

Table S4. Kennedy's approach applied in AP2.

Calculation table to obtain the data quality indicator of (Kennedy et al. 1996)					
**% Attainable Data Quality (x)**	**Aggregated Data Quality Indicator (I)**	**Shape parameters**		**Extents (% variation from mean)**	
		**α**	**β**	**A**	**B**
100	5	5	5	-10	10
$87.5 \le x < 100$	4.5	4	4	-15	15
$75 \le x < 87.5$	4	3	3	-20	20
$62.5 \le x < 75$	3.5	2	2	-25	25
$50 \le x < 62.5$	3	1	1	-30	30
$37.5 \le x < 50$	2.5	1	1	-35	35
$25 \le x < 37.5$	2	1	1	-40	40
$12.5 \le x < 25$	1.5	1	1	-45	45
$0 \le x < 12.5$	1	1	1	-50	50

Table S5. Results of the variation obtained for AP2 with Kennedy (Kennedy et al. 1996) method.

	Selection of scores for parameter input of the LCA Model						Max and lower bounds	
Input	Reliability	Completeness	Temporal correlation	Geographical correlation	Technological correlation	x	A	B
Bagasse	2	3	2	1	3	30	-40	40
Water	2	3	2	4	3	45	-35	35
Sulfuric acid	2	3	2	4	3	45	-35	35
Lime	2	3	2	4	3	45	-35	35
Hydrogen	2	3	2	4	3	45	-35	35
Raney-nickel	2	3	2	4	3	45	-35	35
Electricity	2	3	2	4	3	45	-35	35
Steam	2	3	2	4	3	45	-35	35

For the definition of AP3, published information was used as shown in Table S6 and Equation S1 (Frischknecht et al. 2005).

Table S6. Coefficients used in AP3.

**Indicator score**	**1**	**2**	**3**	**4**	**5**
Reliability	1.00	1.05	1.10	1.20	1.50
Completeness	1.00	1.02	1.05	1.10	1.20
Temporal correlation	1.00	1.03	1.10	1.20	1.50
Geographical correlation	1.00	1.01	1.02		1.10
Further technological correlation	1.00		1.20	1.50	2.00
Sample size	1.00	1.02	1.05	1.10	1.20

The Equation S1 was then used to convert the values from Table S6 to the geometric standard deviation presented in the paper (Table 1) for the AP3.

$$SD_{g95} = \sigma_g^2 = exp^{\sqrt{[ln\,(U_1)]^2+[ln\,(U_2)]^2+[ln\,(U_3)]^2+[ln\,(U_4)]^2+[ln\,(U_5)]^2+[ln\,(U_b)]^2}} \quad (S1)$$

Where U1 is the uncertainty factor of reliability, U2 is the uncertainty factor of completeness, U3 is the uncertainty factor of temporal correlation, U4 is the uncertainty factor of geographic correlation, U5 is the uncertainty factor of other technological correlation, U6 is the uncertainty factor of sample size, and Ub is the basic uncertainty factor.

Table S7. Ecoinvent factors (a_k)

Indicator	Unit	Bagasse	Water	Sulfuric acid	Lime	Hydrogen	Raney-nickel	Electricity	Steam
GWP	kg $CO_{2\ eq}$	3.63E-02	1.35E-03	1.64E-01	1.19E+00	1.61E+00	1.75E+01	2.71E-01	3.37E-01
ODP	kg $CFC11_{eq}$	1.00E-07	0.00E+00	1.00E-07	1.00E-07	2.60E-06	1.55E-05	1.60E-06	1.00E-07
IRP	kBq Co-60 $_{eq}$	8.31E-04	3.27E-05	1.13E-02	8.77E-03	8.50E-02	2.69E+00	3.05E-03	2.97E-03
HOFP	kg $NO_{x\ eq}$	9.39E-05	3.20E-06	1.01E-03	7.70E-04	5.74E-03	7.86E-02	1.26E-03	4.41E-04
PMFP	kg $PM_{2.5\ eq}$	9.04E-05	3.30E-06	2.31E-03	4.03E-04	4.58E-03	4.52E-01	9.09E-04	3.25E-04
EOFP	kg $NO_{x\ eq}$	9.55E-05	3.30E-06	1.03E-03	7.91E-04	6.08E-03	8.01E-02	1.30E-03	4.52E-04
TAP	kg $SO_{2\ eq}$	1.81E-04	4.60E-06	7.37E-03	9.85E-04	1.37E-02	1.53E+00	2.66E-03	8.72E-04
FEP	kg P_{eq}	1.94E-05	7.00E-07	3.15E-04	1.48E-04	1.27E-03	1.78E-02	9.34E-05	5.83E-05
MEP	kg N_{eq}	8.60E-06	1.00E-07	8.40E-06	9.60E-06	7.00E-06	2.06E-03	3.79E-04	2.80E-06
TETP	kg 1,4-DCB	1.49E-01	2.50E-03	2.56E+01	1.98E+00	6.09E+00	2.20E+03	1.06E+00	1.16E+00
FETP	kg 1,4-DCB	3.05E-03	4.22E-05	2.25E-01	2.29E-03	1.09E-02	9.76E+00	1.84E-02	2.16E-03
METP	kg 1,4-DCB	4.75E-03	5.78E-05	2.93E-01	4.50E-03	2.25E-02	1.32E+01	1.27E-02	3.52E-03
HTPc	kg 1,4-DCB	2.62E-03	4.37E-04	4.44E-02	6.20E-03	4.15E-02	2.66E+00	1.01E-02	4.41E-03
HTPnc	kg 1,4-DCB	4.32E-02	1.34E-03	4.23E+00	9.73E-02	3.79E-01	1.77E+02	6.86E-01	1.03E-01
LOP	m^2a crop eq	3.58E-03	1.91E-05	1.49E-02	1.82E-02	1.30E-01	6.72E-01	4.09E-01	2.85E-03
SOP	kg Cu eq	1.80E-04	5.90E-06	1.44E-02	1.68E-04	1.82E-03	5.48E+00	1.24E-03	8.15E-05
FFP	kg oil eq	1.02E-02	3.57E-04	6.41E-02	1.13E-01	3.24E+00	5.08E+00	2.75E-02	9.89E-02
WCP	m^3	7.26E-02	1.01E-03	1.38E-02	7.27E-04	2.35E-03	1.75E+00	5.91E-02	4.07E-04

Table S8. Uncertainty propagation for AP1.

Indicator	Unit	Total	Total (MC)	SD	Error
GWP	kg $CO_{2\ eq}$	8,62E+00	8,45E+00	5,02E-01	-1,94E-02
ODP	kg $CFC11_{eq}$	4,68E-06	4,58E-06	2,65E-07	-2,20E-02
IRP	kBq Co-60 $_{eq}$	1,93E-01	1,89E-01	1,11E-02	-2,04E-02
HOFP	kg $NO_{x\ eq}$	1,55E-02	1,51E-02	8,93E-04	-2,00E-02
PMFP	kg $PM_{2.5\ eq}$	2,85E-02	2,80E-02	1,64E-03	-2,04E-02
EOFP	kg $NO_{x\ eq}$	1,58E-02	1,55E-02	9,14E-04	-2,00E-02
TAP	kg $SO_{2\ eq}$	8,96E-02	8,78E-02	5,15E-03	-2,05E-02
FEP	kg P_{eq}	2,62E-03	2,57E-03	1,55E-04	-1,80E-02
MEP	kg N_{eq}	7,24E-04	7,08E-04	4,07E-05	-2,27E-02
TETP	kg 1,4-DCB	1,43E+02	1,40E+02	8,48E+00	-1,72E-02
FETP	kg 1,4-DCB	7,70E-01	7,59E-01	4,72E-02	-1,38E-02
METP	kg 1,4-DCB	1,03E+00	1,02E+00	6,31E-02	-1,40E-02

HTPc	kg 1,4-DCB	3,03E-01	2,97E-01	1,80E-02	-1,77E-02
HTPnc	kg 1,4-DCB	1,57E+01	1,54E+01	9,56E-01	-1,44E-02
LOP	m^2a crop eq	6,79E-01	6,64E-01	3,84E-02	-2,21E-02
SOP	kg Cu eq	2,33E-01	2,28E-01	1,33E-02	-2,14E-02
FFP	kg oil eq	2,30E+00	2,25E+00	1,31E-01	-2,13E-02
WCP	m^3	9,75E-01	9,53E-01	5,51E-02	-2,26E-02

Table S9. Uncertainty propagation for AP2.

Indicator	Unit	Total	Total (MC)	SD	Error
GWP	kg $CO_{2\ eq}$	8,62E+00	8,59E+00	1,25E+00	-3,02E-03
ODP	kg CFC11 $_{eq}$	4,68E-06	4,66E-06	6,89E-07	-3,06E-03
IRP	kBq Co-60 $_{eq}$	1,93E-01	1,92E-01	2,81E-02	-3,02E-03
HOFP	kg $NO_{x\ eq}$	1,55E-02	1,54E-02	2,25E-03	-3,03E-03
PMFP	kg $PM_{2.5\ eq}$	2,85E-02	2,85E-02	4,15E-03	-3,02E-03
EOFP	kg $NO_{x\ eq}$	1,58E-02	1,58E-02	2,31E-03	-3,03E-03
TAP	kg $SO_{2\ eq}$	8,96E-02	8,93E-02	1,30E-02	-3,01E-03
FEP	kg P $_{eq}$	2,62E-03	2,61E-03	3,83E-04	-3,03E-03
MEP	kg N $_{eq}$	7,24E-04	7,22E-04	1,07E-04	-3,05E-03
TETP	kg 1,4-DCB	1,43E+02	1,43E+02	2,07E+01	-3,01E-03
FETP	kg 1,4-DCB	7,70E-01	7,68E-01	1,12E-01	-3,02E-03
METP	kg 1,4-DCB	1,03E+00	1,03E+00	1,50E-01	-3,02E-03
HTPc	kg 1,4-DCB	3,03E-01	3,02E-01	4,43E-02	-3,04E-03
HTPnc	kg 1,4-DCB	1,57E+01	1,56E+01	2,27E+00	-3,01E-03
LOP	m^2a crop eq	6,79E-01	6,76E-01	9,89E-02	-3,03E-03
SOP	kg Cu eq	2,33E-01	2,32E-01	3,37E-02	-3,00E-03
FFP	kg oil eq	2,30E+00	2,29E+00	3,34E-01	-3,02E-03
WCP	m^3	9,75E-01	9,72E-01	1,57E-01	-3,34E-03

Table S10. Uncertainty propagation for AP3.

Indicator	Unit	Total	Total (MC)	SD	Error
GWP	kg $CO_{2\ eq}$	8,62E+00	8,80E+00	1,19E+00	2,06E-02
ODP	kg CFC11 $_{eq}$	4,68E-06	4,77E-06	5,03E-07	1,95E-02
IRP	kBq Co-60 $_{eq}$	1,93E-01	1,97E-01	2,35E-02	1,95E-02
HOFP	kg $NO_{x\ eq}$	1,55E-02	1,58E-02	1,70E-03	2,02E-02
PMFP	kg $PM_{2.5\ eq}$	2,85E-02	2,91E-02	3,74E-03	1,93E-02
EOFP	kg $NO_{x\ eq}$	1,58E-02	1,61E-02	1,74E-03	2,02E-02
TAP	kg $SO_{2\ eq}$	8,96E-02	9,13E-02	1,24E-02	1,92E-02
FEP	kg P $_{eq}$	2,62E-03	2,67E-03	2,63E-04	2,00E-02
MEP	kg N $_{eq}$	7,24E-04	7,38E-04	1,03E-04	1,90E-02
TETP	kg 1,4-DCB	1,43E+02	1,46E+02	1,89E+01	1,93E-02
FETP	kg 1,4-DCB	7,70E-01	7,85E-01	9,79E-02	1,93E-02

METP	kg 1,4-DCB	1,03E+00	1,05E+00	1,31E-01	1,93E-02
HTPc	kg 1,4-DCB	3,03E-01	3,09E-01	2,92E-02	1,98E-02
HTPnc	kg 1,4-DCB	1,57E+01	1,60E+01	1,84E+00	1,94E-02
LOP	m^2a crop eq	6,79E-01	6,91E-01	1,08E-01	1,88E-02
SOP	kg Cu eq	2,33E-01	2,37E-01	4,27E-02	1,88E-02
FFP	kg oil eq	2,30E+00	2,34E+00	3,42E-01	2,07E-02
WCP	m^3	9,75E-01	9,95E-01	1,55E-01	2,06E-02

References

Al R, Behera CR, Zubov A, et al (2019) Meta-modeling based efficient global sensitivity analysis for wastewater treatment plants – An application to the BSM2 model. Comput Chem Eng 127:233–246. https://doi.org/10.1016/j.compchemeng.2019.05.015

Bacatelo M, Capucha F, Ferrão P, et al (2024) Life cycle assessment of synthetic natural gas production from captured cement's CO2 and green H2. Journal of CO2 Utilization 83:102774. https://doi.org/10.1016/j.jcou.2024.102774

Bamber N, Turner I, Arulnathan V, et al (2020) Comparing sources and analysis of uncertainty in consequential and attributional life cycle assessment: review of current practice and recommendations. Int J Life Cycle Assessment 25:168–180

Ciroth A, Muller S, Weidema B, Lesage P (2016) Empirically based uncertainty factors for the pedigree matrix in ecoinvent. Int J Life Cycle Assessment 21:1338–1348. https://doi.org/10.1007/s11367-013-0670-5

Cucurachi S, Borgonovo E, Heijungs R (2016) A Protocol for the Global Sensitivity Analysis of Impact Assessment Models in Life Cycle Assessment. Risk Analysis 36:357–377. https://doi.org/10.1111/risa.12443

Di Lullo G, Gemechu E, Oni AO, Kumar A (2020) Extending sensitivity analysis using regression to effectively disseminate life cycle assessment results. Int J Life Cycle Assess 25:222–239. https://doi.org/10.1007/s11367-019-01674-y

Edelen A, Ingwersen W (2016) Guidance on Data Quality Assessment for Life Cycle Inventory Data

Fatehi P, Catalan L, Cave G (2014) Simulation analysis of producing xylitol from hemicelluloses of pre-hydrolysis liquor. Chem Eng Res Des 92:1563–1570. https://doi.org/10.1016/j.cherd.2014.03.010

Frischknecht R, Jungbluth N, Althaus HJ, et al (2005) The ecoinvent Database: Overview and Methodological Framework. Int J Life Cycle Assessment 10:3–9

Geisler G (2003) ife Cycle Assessment in the Development of Plant Protection Products: Methodological Improvements and Case Study. Swiss Federal Institute of Technology (ETH)

Geisler G, Hellweg S, Hungerbühler K (2005) Uncertainty Analysis in Life Cycle Assessment (LCA): Case Study on Plant - Protection Products and Implications for Decision Making (3 pp). Int J Life Cycle Assess 10:192.1-192.3. https://doi.org/10.1065/lca2004.09.178.1

Geisler G, Hofstetter TB, Hungerbühler K (2004) Production of fine and speciality chemicals: procedure for the estimation of LCIs. Int J Life Cycle Assess 9:101–113. https://doi.org/10.1007/BF02978569

Golsteijn L (2015) Behind the scenes at Monte Carlo simulations. In: https://pre-sustainability.com/articles/behind-the-scenes-at-monte-carlo-simulations/

Groen EA, Bokkers EAM, Heijungs R, de Boer IJM (2017) Methods for global sensitivity analysis in life cycle assessment. Int J Life Cycle Assess 22:1125–1137. https://doi.org/10.1007/s11367-016-1217-3

Groen EA, Heijungs R, Bokkers EAM, De Boer IJM (2014) Sensitivity analysis in life cycle assessment. In: Proceed 9th Int Conf Life Cycle Assess Agrifood Sec

Heijungs R, Lenzen M (2014) Error propagation methods for LCA - A comparison. Int J Life Cycle Assessment 19:1445–1461. https://doi.org/10.1007/s11367-014-0751-0

Heijungs R, Suh S (2002) The Computational Structure of Life Cycle Assessment. Springer Netherlands, Dordrecht

Herman J, Usher W (2017) SALib: An open-source Python library for Sensitivity Analysis. J Open Source Softw 2:97. https://doi.org/10.21105/joss.00097

Igos E, Benetto E, Meyer R, et al (2019) How to treat uncertainties in life cycle assessment studies? Int J Life Cycle Assessment 24:794–807. https://doi.org/10.1007/s11367-018-1477-1

ISO (2006a) ISO 14044:2006 Environmental management Life cycle assessment Requirements and guidelines

ISO (2006b) ISO 14040: 2006 Environmental management Life cycle assessment Principles and framework. https://www.iso.org/standard/37456.html

Karka P, Papadokonstantakis S, Kokossis A (2017) Cradle-to-gate assessment of environmental impacts for a broad set of biomass-to-product process chains. Int J Life Cycle Assess 22:1418–1440. https://doi.org/10.1007/s11367-017-1262-6

Kennedy DJ, Montgomery DC, Quay BH (1996) Data quality. Int J Life Cycle Assess 1:199–207. https://doi.org/10.1007/BF02978693

Lloyd SM, Ries R (2007) Characterizing, propagating, and analyzing uncertainty in life-cycle assessment: A survey of quantitative approaches. J Ind Ecol 11:161–179. https://doi.org/10.1162/jiec.2007.1136

Özüdoğru HMR (2018) Techno-economic Analysis of Xylitol, Citric Acid and Glutamic Acid Biorefinery Scenarios Utilizing Sugarcane Lignocellulose

Paiva JE de, Maldonade IR, Scamparini ARP (2009) Xylose production from sugarcane bagasse by surface response methodology. Revista Brasileira de Engenharia Agrícola e Ambiental 13:75–80. https://doi.org/10.1590/S1415-43662009000100011

Qiao Y, Wen X, Liu S, et al (2025) Stochastic analysis for comparing life cycle carbon emissions of hot and cold mix asphalt pavement systems. Resour Conserv Recycl 212:107881. https://doi.org/10.1016/j.resconrec.2024.107881

Qin Y, Cucurachi S, Suh S (2020) Perceived uncertainties of characterization in LCA: a survey. Int J Life Cycle Assess 25:1846–1858. https://doi.org/10.1007/s11367-020-01787-9

Reed PM, Hadjimichael A, Malek K, et al (2022) Addressing uncertainty in multisector dynamics research. Zenodo

Ross SA, Cheah L (2019) Uncertainty Quantification in Life Cycle Assessments: Exploring Distribution Choice and Greater Data Granularity to Characterize Product Use. J Ind Ecol 23:335–346. https://doi.org/10.1111/jiec.12742

Saltelli A, Tarantola S, Campolongo F, Ratto M (2004) Sensitivity Analysis in Practice. Wiley

Saltelli A, Tarantola S, Chan KPS (1999) A quantitative model-independent method for global sensitivity analysis of model output. Technometrics 41:39–56. https://doi.org/10.1080/00401706.1999.10485594

Schröder D, Hegner R, Güngör A, Atakan B (2019) The influence of uncertainty of economic parameters and upscaling on product costs of an engine polygeneration system. In: Proceedings of the ECOS 2019. POLAND

Serbouti A, Rattal M, Boulal A, et al (2018) Application of sensitivity analysis and genopt to optimize the energy performance of a building in Morocco. Int J Engineering & Technology 7:2068. https://doi.org/10.14419/ijet.v7i4.13280

Shaji A, Shastri Y, Kumar V, et al (2022) Sugarcane bagasse valorization to xylitol: Techno-economic and life cycle assessment. Biofuels, Bioproducts and Biorefining 16:1214–1226. https://doi.org/10.1002/bbb.2368

Shi R, Guest JS (2020) BioSTEAM-LCA: An Integrated Modeling Framework for Agile Life Cycle Assessment of Biorefineries under Uncertainty. ACS Sustain Chem Eng 8:18903–18914. https://doi.org/10.1021/acssuschemeng.0c05998

Sobol IM (2001) Global sensitivity indices for nonlinear mathematical models and their Monte Carlo estimates. Math Comput Simul 55:271–280. https://doi.org/10.1016/S0378-4754(00)00270-6

Stajić L, Praksová R, Brkić D, Praks P (2024) Estimation of global natural gas spot prices using big data and symbolic regression. Resour Policy 95:105144. https://doi.org/10.1016/j.resourpol.2024.105144

Sun S, Ertz M (2020) Life cycle assessment and Monte Carlo simulation to evaluate the environmental impact of promoting LNG vehicles. MethodsX 7:101046. https://doi.org/10.1016/j.mex.2020.101046

Weidema BP, Suhr M (1996) Data quality management for life cycle inventories-an example of using data quality indicators. J Cleaner Prod 4:167–174. https://doi.org/https://doi.org/10.1016/S0959-6526(96)00043-1

Wei W, Larrey-Lassalle P, Faure T, et al (2015) How to conduct a proper sensitivity analysis in life cycle assessment: Taking into account correlations within LCI data and interactions within the LCA calculation model. Environ Sci Technol 49:377–385. https://doi.org/10.1021/es502128k

Zhang X, Trame M, Lesko L, Schmidt S (2015) Sobol sensitivity analysis: A tool to guide the development and evaluation of systems pharmacology models. CPT: Pharmacom Syst Pharm 4:69–79. https://doi.org/10.1002/psp4.6
